# Distinguishing Abrupt and Gradual Forest Disturbances With MODIS-Based Phenological Anomaly Series

**DOI:** 10.3389/fpls.2022.863116

**Published:** 2022-05-23

**Authors:** Anne Gnilke, Tanja G. M. Sanders

**Affiliations:** ^1^Department of Forest Ecology and Biodiversity, Thünen Institute of Forest Ecosystems, Eberswalde, Germany; ^2^Department of Disturbance Ecology and Vegetation Dynamics, University of Bayreuth, Bayreuth, Germany

**Keywords:** forest disturbance, MODIS, ground-truthing, EVI anomaly, phenological series

## Abstract

Capturing forest disturbances over time is increasingly important to determine the ecosystem's capacity to recover as well as aiding a timely response of foresters. With changes due to climate change increasing the frequencies, a better understanding of forest disturbances and their role in historical development is needed to, on the one hand, develop forest management approaches promoting ecosystem resilience and, on the other hand, provide quick and spatially explicit information to foresters. A large, publicly available satellite imagery spanning more than two decades for large areas of the Earth's surface at varying spatial and temporal resolutions represents a vast, free data source for this. The challenge is 2-fold: (1) obtaining reliable information on forest condition and development from satellite data requires not only quantification of forest loss but rather a differentiated assessment of the extent and severity of forest degradation; (2) standardized and efficient processing routines both are needed to bridge the gap between remote-sensing signals and conventional forest condition parameters to enable forest managers for the operational use of the data. Here, we investigated abiotic and biotic disturbances based on a set of ground validated occurrences in various forest areas across Germany to build disturbance response chronologies and examine event-specific patterns. The proposed workflow is based on the R-package “npphen” for non-parametric vegetation phenology reconstruction and anomaly detection using MODIS EVI time series data. Results show the potential to detect distinct disturbance responses in forest ecosystems and reveal event-specific characteristics. Difficulties still exist for the determination of, e.g., scattered wind throw, due to its subpixel resolution, especially in highly fragmented landscapes and small forest patches. However, the demonstrated method shows potential for operational use as a semi-automatic system to augment terrestrial monitoring in the forestry sector. Combining the more robust EVI and the assessment of the phenological series at a pixel-by-pixel level allows for a changing species cover without false classification as forest loss.

## Introduction

Disturbances play a substantial role in forest ecosystems, influencing the structure of stands, their regeneration, and the character of the whole forest ecosystem (Dale et al., 2001). As such, forest disturbances are an integral part of these ecosystems including storm impact, droughts, flooding, fires, and insect or disease outbreaks in plants and animals. Here, we used the term disturbance to describe any negative deviation from the long-term enhanced vegetation index (EVI) phenology linked to abiotic or biotic causes. Changes in the severity and frequency of these disturbances, due to an increase in extreme climate events (Seneviratne et al., 2012), raise concerns regarding forest function and provision, thus sparking great interest in large-scale forest assessments (for a list of current remote-sensing-based forest monitoring in Germany refer to Supplementary Material 1). A current increase in disturbance events leads to multiple assessments (Senf and Seidl, 2020). The analysis relies on either remote-sensing products, covering large and continuous scales, as done for mortality (Senf et al., 2018) and land-cover change (Hansen and Loveland, 2012), or on ground assessments such as the ICP Forests Crown Condition Survey (George et al., 2021). In recent years, remote-sensing products have begun to increase the value of ground assessments by being less personnel and resource-demanding, hence producing lower cost and allowing a higher temporal resolution of monitoring cycles (Lausch et al., 2018). Large benefits are the possibility to redo the analysis and repeat it retrospectively, as well as cover continuous areas rather than individual sampling plots in the analysis. Despite the advantages, remote-sensing products and applications alone do not provide all the answers needed (Chávez et al., 2019) in the current forest monitoring and practice seemingly leading to a limited acceptance of foresters. Thus, a workflow for retrieving up-to-date, reliable and precise information on the forest vitality status from satellite data is of great interest for early stage warning and diagnosis, as well as to support in preventative- and countermeasures in today's forest practice (Torresan et al., 2021). However, disturbances in forests lead to a variety of symptoms, from the loss or discoloration of leaves and needles (e.g., insects, fungi, drought) and the disruption of forest structures to the loss of standing wood volume (e.g., storm, fire) (Buma, 2015), which are so far assessed individually (Gao et al., 2020). Providing this information in a single, easy-to-use workflow would improve the usability of forest practice. This is specifically relevant in fast-changing conditions due to regeneration or in forest areas repeatedly affected by biotic or abiotic disturbances (Iglhaut et al., 2019). Therefore, foresters and decision-makers need a spatially explicit and timely assessment of the disturbance, with information on the disturbance history to assess the vulnerability of existing stands and the cause of the disturbance.

During the last decade, several extreme weather events contributed to considerable loss and degradation of forest ecosystems throughout central Europe and Germany (Oeser et al., 2017). With various extreme events likely to increase in the future (Seneviratne et al., 2012), the risk of single and multiple disturbance events increases further. However, the different causes, roughly classified as biotic and abiotic, lead to different disturbance patterns and require distinct silvicultural measures. While water stress and drought are associated as the triggering factors for early defoliation, vitality loss, and forest pathogens (Ghelardini et al., 2017; Bußkamp et al., 2020), water stress is linked to increased vulnerability due to fungal infestation (Blodgett et al., 1997; Fabre et al., 2011). While water-related disturbances vary between site conditions and species strategies, other abiotic disturbances such as fire and storm act at a larger level, which is less tightly linked to these disturbances. However, even here, the detection of the disturbance is increased by the knowledge of forest cover. Windstorms are widely referred to as abiotic and temporally discrete events and are due to their often spatially explicit occurrence that is operationally detectable (Giannetti et al., 2021). The destructive potential of a storm event depends on the (peak) wind speeds, the duration, and the frequency with which forest stands are affected. The severity of storm-related impact on a given forest stand depends on the stocked species, species admixture, and age (Valinger and Fridman, 2011). In combination with the precipitation, the severity and extent of storm-induced destruction as well as mortality rates increase in the forest stand level (Usbeck et al., 2010). Forest fires are commonly classified as temporally discrete, abiotic disturbance events that result in abrupt changes in the form of burned vegetation and soil (Gnilke and Sanders, 2021). In Germany, forest fires usually occur in the summer period from April to August. The spread of forest fire is influenced by meteorological conditions and topography as well as forest and site characteristics (White et al., 1996). Forest areas affected by fire usually consist of a single contiguous burn area or a few compactly shaped burned areas that are locally concentrated. Within the burned areas, a zonal gradation of fire severity can be observed, which typically decreases toward the outer edges. Insects and fungi on the other hand are less pronounced during events that can occur from single trees to larger stands (Kautz et al., 2018). Here, this differentiation between abrupt and gradual changes allows the first differentiation between abiotic and biotic changes, which remained difficult as described in the study of Senf and Seidl (2021) and Francini et al. (2022). So far, a combination of remote-sensing data and models is used to map abiotic disturbances by fire and storm. The examples presented in this study showed that the non-parametric method of the analysis of Moderate Resolution Imaging Spectroradiometer (MODIS)-derived phenological series and the self-calibrating change detection allows the classification of multiple disturbances without relying on pre-assumptions or the need of user-defined thresholds for curve fitting or trend modeling. Abrupt changes can be further analyzed regarding their size, distribution, amplitude of change, and recovery. This allows a determination of disturbances caused by fire or storm limited only by the resolution of the used remote-sensing product.

Over the years, a variety of remote-sensing platforms are developed with specific or overlapping sensors, with the most commonly used ones being optical imaging systems (for more information, refer to Lechner et al., 2020). To obtain information on forest structure, vitality, or loss, to name just a few, Landsat, MODIS, and sentinel data are used (McDowell et al., 2015). While Landsat provides the longest time series and a high spatial resolution offering information on a small scale or on gradually occurring disturbances (DeVries et al., 2015); however, it still relies on potent computing power and algorithms, e.g., machine-learning classifiers, and lacks in prepossessing and validation (Banskota et al., 2014), as well as has low temporal resolution. MODIS detection accuracy lies in agreement with Landsat (Jin and Sader, 2005) and provides 20 years of prepossessed data in high temporal resolution, and only two scenes cover the entire part of Germany. Sentinel data provide higher spatial-spectral and radiometric resolution, which are ready-to-use products; however, the time series is still short but will provide ample information in the future. Nevertheless, several studies use Sentinel data to detect current changes (Puletti et al., 2019; Senf and Seidl, 2021). All three provide passive optical/NIR and mid-infrared information on the surface reflectance, which allows the application of conventional and innovative vegetation indices and land cover applications. From all these, the mission's spectral vegetation indices (VI), such as the Normalized Difference Vegetation Index (NDVI) and the Enhanced Vegetation Index (EVI), can be deducted and are widely used to assess vegetation conditions and monitor ecological phenomena and their dynamics across various spatial and temporal scales (Bannari et al., 1995; Myneni et al., 1995; Hunt et al., 2011; Decuyper et al., 2020; Schuldt et al., 2020). From these indices, phenological series can be derived, providing the start of the vegetation period by greening and the end by the loss of greenness. While the start and the end of the vegetation period vary slightly between the years, the general curve matches and can therefore be used to detect changes. With a good agreement of the phenological series between MODIS and Sentinel 2 (Thapa et al., 2021), the use of both is possible, and also, the longer time series of MODIS provides a reliable reference curve, whereas Landsat and Sentinel 2 lack the temporal resolution and face occlusions due to cloud coverages in more humid latitudes. Currently, however, even the combination of multiple sources of remote sensing is possible and could increase the information content further (Lu et al., 2016; Wan et al., 2021). Limits, however, do occur in the processing capacity, the data handling, and the interpretation (Arvor et al., 2019).

This need for methodological fine-tuning is met by a huge variety of disturbances. Disturbance events can cause abrupt or gradual changes, showing distinct or non-distinct patterns in space and time (Sturtevant and Fortin, 2021). These patterns are further modified by an overlay of different disturbances (Cannon et al., 2017) resulting in additive effects, influencing the expected patterns, and making the recognition and differentiation more difficult. Small scale and patchy disturbances can retain younger and remnant live trees aiding recovery (Seidl et al., 2014) but also mask the signal received limiting interpretation. The scale of the disturbance will not only influence the recovery potential but also influence the ability to detect it. Storms generally leave younger and remnant trees, whereas fires, especially with high temperatures and in monocultures, can leave nothing behind, making them easy to detect. Another important information is that the severity of the disturbance is linked to the spatial and temporal extent, the severity, and the frequency (Hart and Kleinman, 2018). All these processes will influence the likeliness of detection and therefore need to be considered. The analysis of time series adds the temporal dimension and is therefore an important source of information for the extraction of semantic content from satellite scenes (Zhang et al., 2006; Atzberger et al., 2014; Recuero et al., 2019). Hence, time series analysis methods are useful to efficiently spot forest vitality decline at an early stage, which is often essential to effectively perform damage control and mitigate long-term effects in forests by adaptive forest management (Bolte et al., 2009).

Despite the successful application at local to regional scales, results often show huge variances between the ground estimates and the remote-sensing assessments, leading to a lack of acceptance in foresters and politicians. To overcome these issues, a substantiated validation of the remote-sensing results with reliable ground data is the key factor (Frolking et al., 2009). While we are convinced that remote sensing is an important addition to ground-based monitoring systems, we believe that methods need to be robust to bridge data gaps that occur, e.g., cloud cover, independent of artificial thresholds for trend modeling or curve fitting and to be ready to use across various forest cover types and growth conditions. In addition, they need to be useable by a non-specialist and without high computer performance while still providing reliable information. Here, we used a self-calibrating non-parametric approach for time series analysis of MODIS EVI data for detecting and assessing various disturbed areas in Germany observed over the last 20 years to improve the understanding of disturbance regimes in forest ecosystem to (a) detect patterns of disturbance, (b) capture distinct features determining abrupt and gradual change, and (c) assess their magnitudes of severity, as well as (d) disturbance interactions.

## Materials and Methods

**Figure 1:**
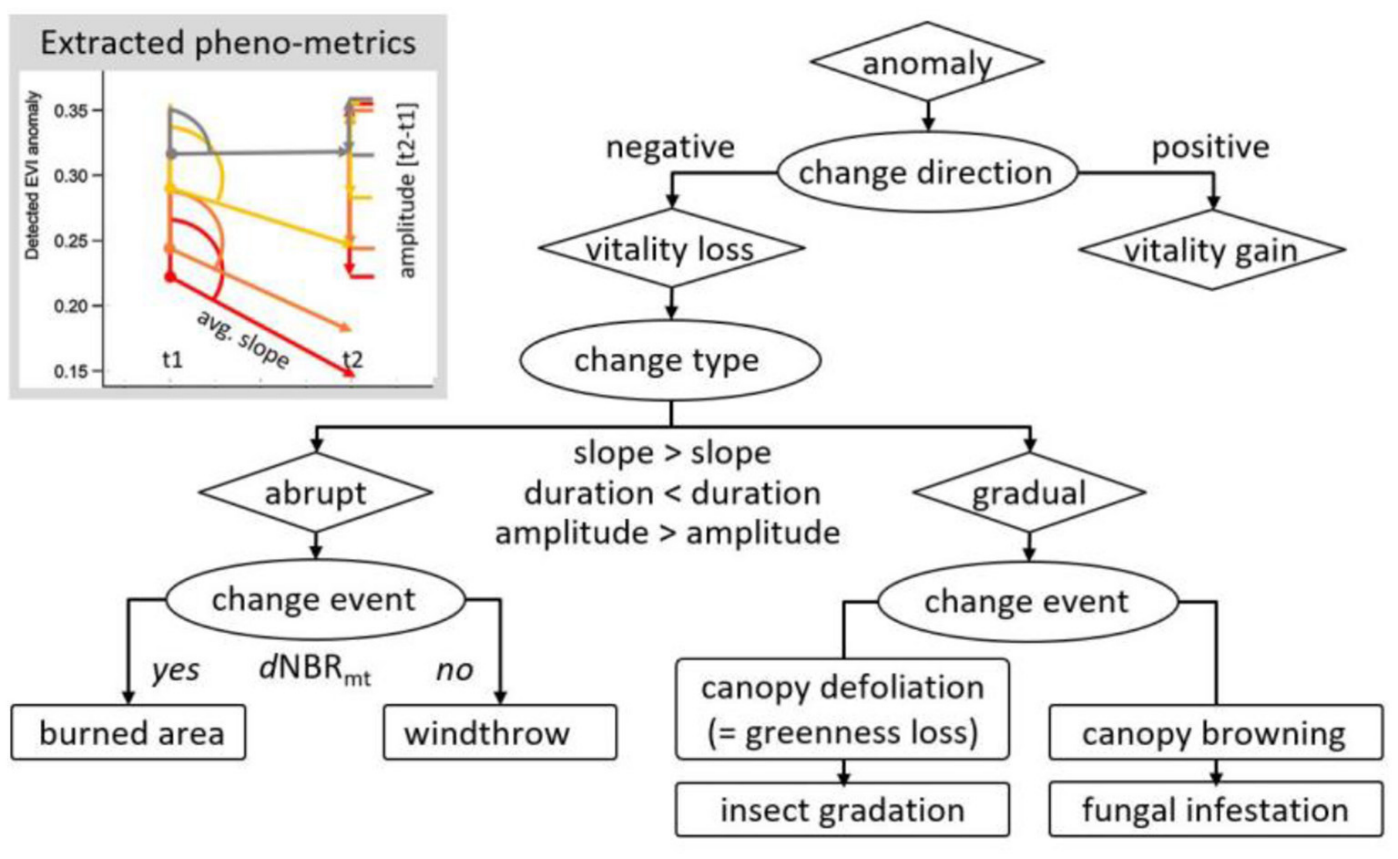
Schematic presentation of the differentiation between the different disturbance types using the parameters slope, duration, and amplitude to first distinguish between abrupt and gradual change, defined by the duration of the decline and then to distinguish between the cause for the change event.

**Figure 2:**
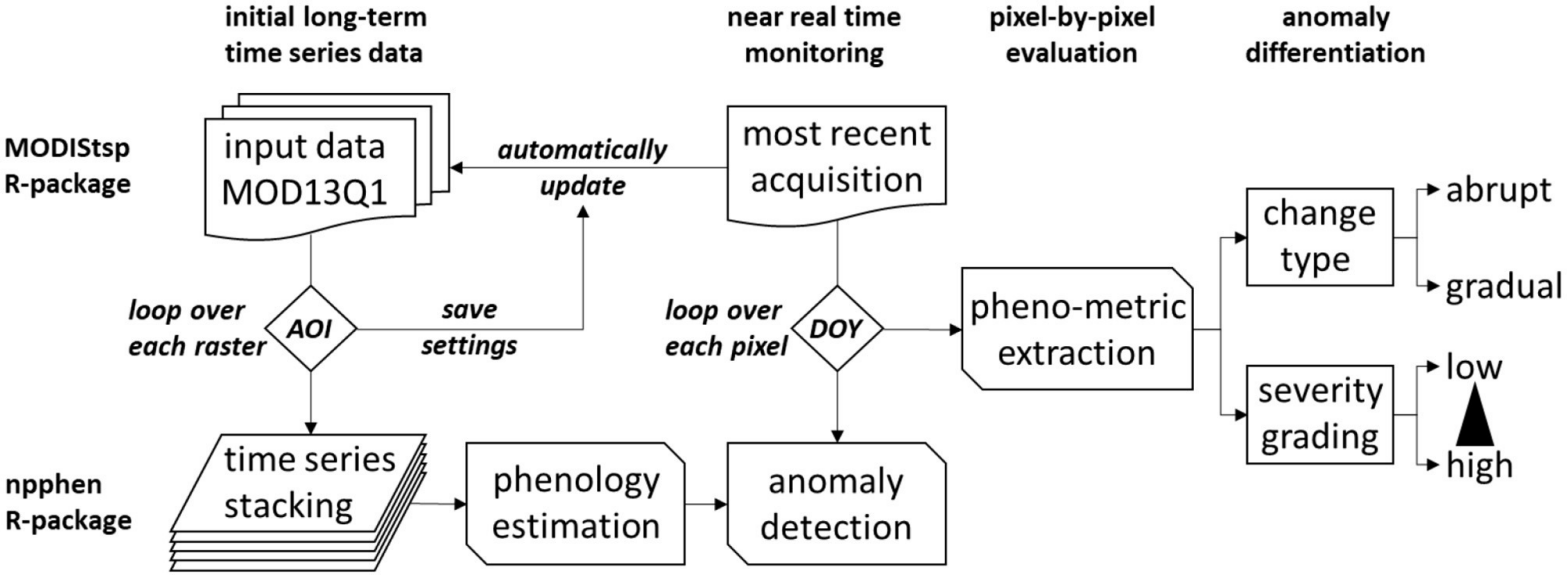
Proposed workflow to automatically extract disturbances.

Disturbance-specific features seen as phenological metrics, such as duration, slope, and amplitude, were used to deduce the timing and quantify the abruptness and the severity of the deviation relative to the long-term normal (estimated EVI phenology vs. expected EVI) (Jönsson and Eklundh, 2004). These prototypes are used to create a multistage, rule-based decision tree for disturbance-specific pixel classification (Figures 1, 2). While the duration describes the temporal factor over which the disturbance response evolves, starting from the point in time when the deviation of observed EVI values from the long-term EVI phenology first occurs, where the amplitude defines the drop of the EVI anomaly, and the slope is a combination of these two factors. The utilization of additional remote sensing products, e.g. multi-temporal differenced normalized burn ratio (dNBR) (Veraverbeke et al., 2011) could further improve the distinction between different disturbance types and their potential causes. However, there is still a limitation regarding how to quantify the magnitude of severity, which currently is not comparable between different sites easily but does rather provide a grading within each investigated site of this study. In the next step, these disturbance patterns could be exploited as disturbance markers to detect similar patterns within the EVI chronologies of the last 20 years potentially across multiple pixels. However, the problem of interpreting spectral time series signatures of irreversible damage changing forest cover type, e.g., from coniferous forest to mixed or deciduous broad-leaf forest remains. However, the kernel density estimation (KDE)-based phenological reconstruction algorithm is based on data inherent thresholds (non-parametric), and thus, it follows a robust statistical approach not relying on any artificial or given thresholds. It can be applied at any multispectral pixel and across various vegetation cover types or biomes allowing high flexibility in application. The pixel-by-pixel approach is further robust to missing data, e.g., data gaps at any given pixel position within the analyzed sequence. Therefore, no gap filling in the sense of spatial or temporal interpolation is needed to reduce uncertainty in not masking highly dynamic or small-scale phenomena (Figure 2).

### Reference Data

**Figure 3:**
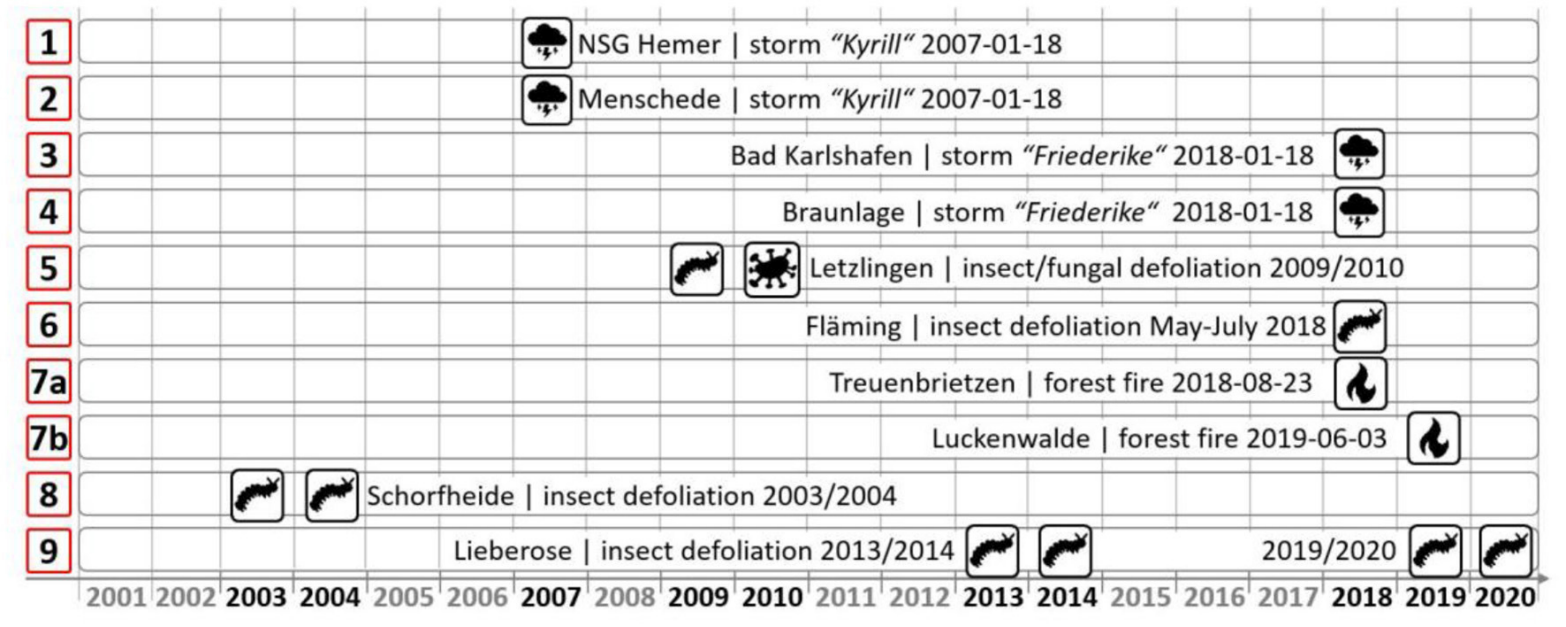
Timeline from 2001 to 2020 indicating the known disturbances used for detection validation of the investigated sites with the site name and the event and on the left the study site numbers for further reference.

Regular reporting based on ground assessments from the federal states provided data on known disturbances caused by fire, storm, insects, fungi, or a combination of the latter two (Figure 3). From these events, nine sites were selected across Germany with disturbances differing in timing, extent (Figure 3, examples 1 and 2), and type of the disturbance. Over the last 20 years, while some sites were reported as only being affected one time (examples 1, 2, 3, 4, and 6), others show a combination of two different disturbances (5) or one disturbance agent occurred multiple times (8, 9). The forest fire sites (7) are in close spatial proximity and should therefore be treated as one.

The reference data for the windthrow areas caused by the storm *Kyrill* (study sites 1 and 2) in North Rhine-Westphalia consist of polygon shapefiles (https://www.opengeodata.nrw.de/produkte/umwelt_klima/wald_forst/wald/windwurfschadflaechen-kyrill_EPSG25832_Shape.zip) mapped within the post-storm forest damage assessment, which was commissioned by the State Forestry and Timber Agency immediately after the storm event. The windthrow delineation was compiled of data from on-site inspections on the ground and aerial color-infrared image interpretation. The reference data for the windthrow areas in North Rhine-Westphalia and Lower Saxony caused by the storm *Friederike* (study sites 3 and 4) consist of Delineation Products created by the Copernicus Emergency Management Service (EMS) (https://emergency.copernicus.eu/mapping/list-of-components/EMSR266) through visual interpretation of satellite images.

The disturbance reference data for the burned areas caused by two major forest fires near Treuenbrietzen and Luckenwalde in South Brandenburg (study sites 7a and 7b) consist of Grading Products created by the Copernicus Emergency EMS (https://emergency.copernicus.eu/mapping/list-of-components/EMSR307) through supervised automatic classification of multi-temporal satellite imagery (Joubert-Boitat et al., 2020). The grading maps show the extent of the burned area and the magnitude of damage referring to the Copernicus EMS severity classes, ranging from “possibly damaged” and “damaged” to “destroyed.”

The data sources used as references for gradual disturbances are based on various forest monitoring reports published by federal institutions and forestry authorities. The study on the local effects of climate change on forestry in selected regions of Saxony-Anhalt conducted by the North-West German Forest Research Institute (NW-FVA) served as a preliminary source of defoliation caused by pine sawfly (*Diprion pini*) and canopy browning due to fungal infestation (*Diplodia* pinea) in the Colbitz-Letzlinger Heide (study site 5). Forest condition reports (https://mluk.brandenburg.de/cms/media.php/lbm1.a.3310.de/Waldzustandsbericht_BB_2018.pdf) and silvicultural monitoring data for nun moth (*Lymantria monacha*) defoliation published by the Brandenburg State Forestry Office (LFE) served as references for the insect-induced disturbances in the Schorfheide (study site 8) and the Lieberoser Heide (study site 9). The grading product indicating the extent and severity in the Fläming region (study site 6) has been created from the Forest Condition Index based on multi-temporal satellite imagery.

**Figure 4:**
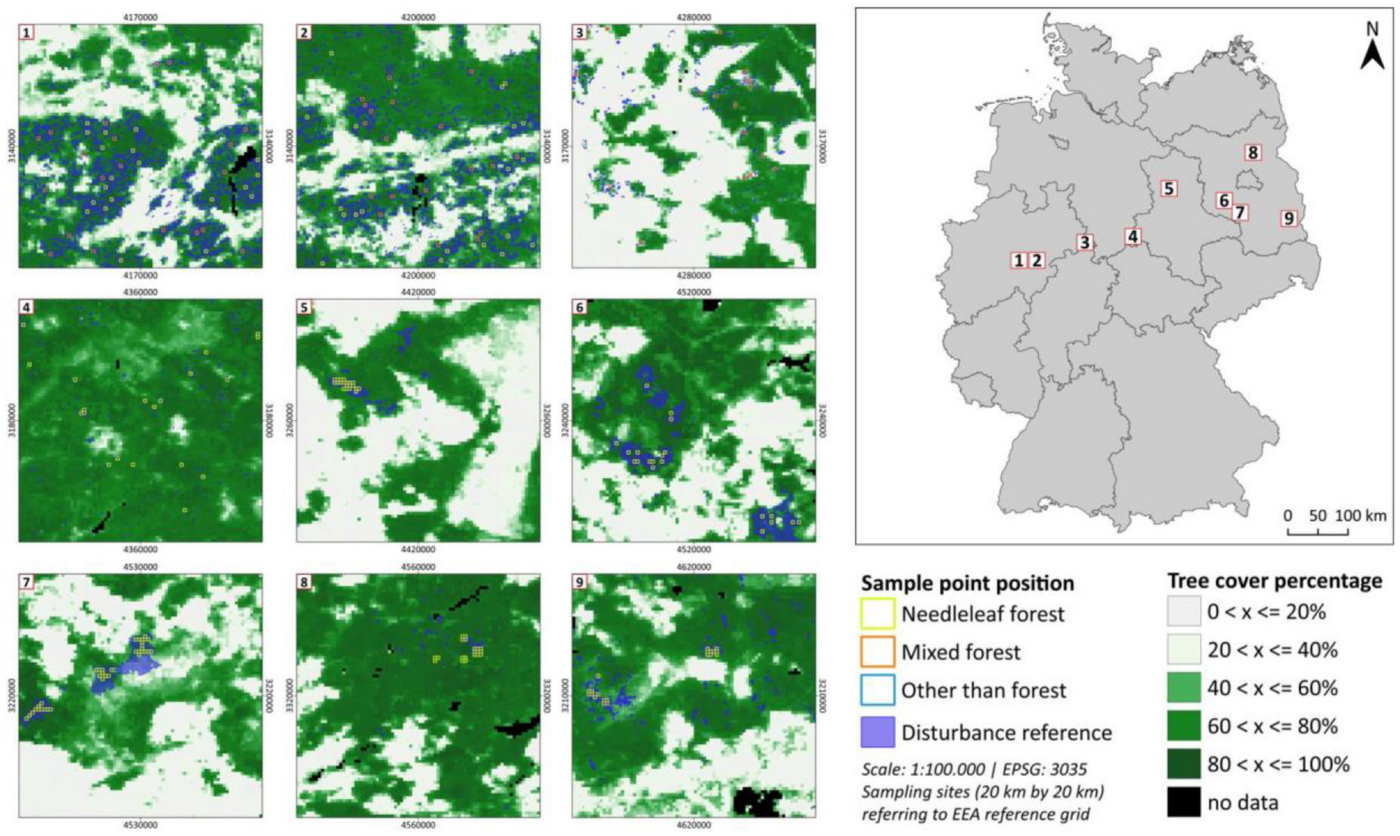
Location of the test-investigated sites across North-West and North-East Germany (right) and percentage tree cover with the sample point position and the disturbance reference for each study site (left) (for a higher resolution please refer to Supplementary Material 2).

The designated study areas, each with an extent of 20 km by 20 km, were created based on the “German Geographic Reference Grid” (Geographical grid for Germany in Lambert projection - Data Europa EU, 2022; BKG; http://data.europa.eu/88u/dataset/02a7e63d-caaa-4ded-b6ff-1f1e73faf883) using the 10 by 10 km grid based on the Lambert Azimuthal Equal Area (ETRS98 LAEA, EPSG: 3035) projection (Figure 4). The systematic grid is INSPIRE compliant so that statistical facts and semantic information can be evaluated in a temporally and spatially consistent and reproducible manner. Through compatibility with national grid systems and the European Environmental Agency (http://www.eea.europa.eu/data-and-maps/data/ds_resolveuid/D63BFD62-6597-4D5F-BD35-9E06265102E0) reference grid, this approach enables both reproducibility for geostatistical evaluations at the national level and transferability of the proposed methodology to areas of interest outside Germany as well as comparisons across Europe. All disturbance reference data are publicly available and free of charge, a detailed list of the reference data is used, and the associated metadata and maps can be found in Supplementary Material 2. The study sites show some of the most common forest types and structures in Northern Germany.

In some cases (referring to the study sites 5, 6, 8, and 9), the reports only provide geo-addresses from the forestry cadastre assigned to the forestry administrative management units, but there was a lack of accurate geolocation or delineation for the actual disturbance areas. To compensate for this, we used the Global Forest Change data (Hansen et al., 2013) at the respective “Forest loss year” when the disturbances occurred for cross-checking the location information given in the disturbance reporting and to narrow down the extent of the actual damaged area affected by insect defoliation or fungal infestation. However, our aim was to detect the gradual changes requiring more than a binary map of forest loss. Therefore, this method aims to capture forest degradation with explicit magnitudes and their severity over time and space.

### MODIS Data

The satellite time-series data used in this study was composed of MODIS Terra Vegetation Indices 16-day composites at 250 m pixel size (MOD13Q1 VI v006) provided by NASA's Land Processes Distributed Active Archive Centre (LP DAAC). The initial MOD13Q1 data are delivered in HDF-EOS file format, where each acquisition consists of two primary vegetation index layers (NDVI/EVI), two quality assessment layers (indicating VI quality and pixel reliability), and the four surface reflectance layers (corresponding to the red, NIR, blue, and mid-infrared bands). The MOD13Q1 VI are compiled of bi-monthly maximum value composites (MVC) representing the best available pixel value from all acquisitions acquired over the respective 16-day time interval according to the criteria “low clouds,” “low view angle,” and “highest NDVI/EVI value” (Huete et al., 2010; Solano, 2015). All study areas considered are within one MODIS tile referring to the sinusoidal grid tile-ID h18v03. Continuous MOD13Q1 time series data of the past 20 years ranging from the first complete annual cycle available in 2001 to 2020 were considered. The total of 460 MODIS EVI records (23 records for each annual cycle) served as time-series input data for estimating the long-term EVI phenology, on which the reference years of known disturbance events at the individual study site are based and scanned for anomalies. The use of sensor-inherent data for time series preparation and filtering enables a spatial–temporal consistent and reproducible evaluation of statistical facts. To identify forest areas, MODIS auxiliary data were used to incorporate semantic information of the forest type and cover. The MODIS Terra Vegetation Continuous Fields (MOD44B v006) percent tree cover layer (DiMiceli et al., 2021) represents the percent of each pixel covered by tree canopy at 250 m by 250 m pixel size. The data layer of the day of the year (DOY) 65 (=5 March) for the respective target year was used to identify forest areas at each study site. The combined MODIS Terra and Aqua Land Cover Type (MCD12Q1 v006) product (Sulla-Menashe et al., 2019) was used as an auxiliary data layer within the time-series preparation procedure. The Land Cover Type 1 data layer was used to assign the extracted pixels to the forest type classes according to the International Geosphere-Biosphere Program (IGBP) legend and class descriptions to address the seasonality effects of “evergreen coniferous forest,” “deciduous broadleaf forest,” and “mixed forest” (e.g., leaf-on/leaf-off) in the time series pattern analysis.

### Pre-processing

The MODIS time series preparation comprises two consecutive subroutines. The *MODIStsp* package version 1.3.9 developed by Busetto and Ranghetti (2016) was used to automatically compute the spectral indices and quality indicators. Additionally, the Geospatial Data Abstraction Library (GDAL) was used for the translation and filtering of the multi-temporal geospatial raster data (Figure 12).

In detail, the *MODIStsp* widgets were used to query available MODIS products according to predefined parameter settings (refer to Supplementary Material 2) and bulk-download the corresponding MOD13Q1v006 HDF-EOS files from the NASA LP DAAC server. The implemented GDAL functionality was used to extract required layers from the initial HDF files and subsequently re-project, clip, and resample the MODIS vegetation index (VI) products and the related per-pixel MODIS VI Quality Assessment (QA) science datasets.

The preprocessed MOD13Q1v006 data required quality filtering to facilitate time series analysis (Witt et al., 2011). The pixel-wise quality filtering scheme was implemented as proposed by Estay and Chávez (2018). The MODIS VI detailed quality assessment (QA) bands are used to parse the QA-bit information to individual layers, which are used for transcription to customize binary data quality masks. Deviating from the widespread procedure to only keep data above the “lowest quality” criterion (Chave et al., 2019), all VI data are kept as valid to limit data loss. Only pixels assigned to “high” aerosol quantity bits were deleted from the remaining data since EVI is less sensible to atmospheric disturbances. All land pixels without “adjacent clouds” or “mixed clouds” and “snow/ice” or “shadow” are considered to be of acceptable quality for this study (refer to Supplementary Material 2). All pixels that do not match the QA criteria are treated as being unreliable and are discarded from further analysis.

### Post-processing

**Figure 5:**
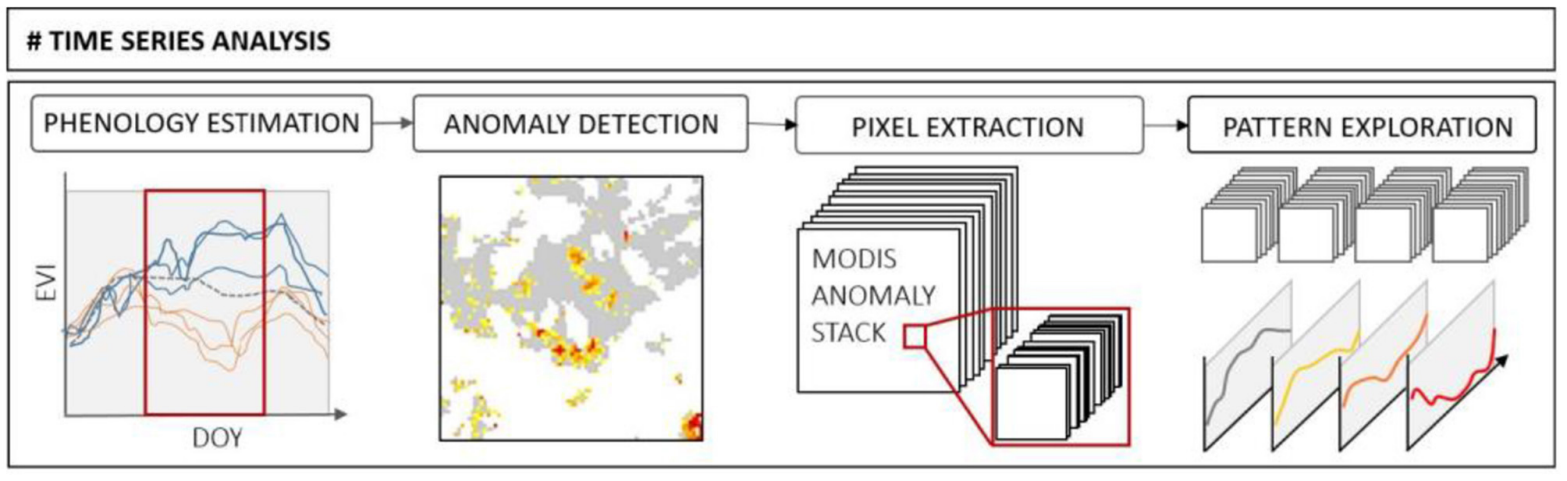
Schematic workflow of the non-parametric time series analysis comprising the subroutines from the kernel density estimation (KDE)-based phenology estimation and anomaly detection to the pixel extraction and pattern exploration.

The non-parametric approach for the phenology estimation and the change detection used in this study was developed by Chávez et al. (2019) and is implemented in the R-package *npphen* (Estay and Chávez, 2018). The algorithm includes two subroutines: the kernel density-based algorithm provides the estimated EVI phenology (EVI_phen_) of the Modis EVI 16-day composite products for the reference period 2001–2020 (Figure 5, phenology estimation).

In the subsequent step, the integrated self-calibrating pixel-by-pixel change detection algorithm is used to identify significant deviations (Figure 5, EVI anomaly) within the EVI signatures observed during the year of disturbance (EVI_obs_) and in relation to the EVI_phen_ curve. The EVI anomaly is calculated using the formula:


$$\begin{array}{l} EVI\;anomaly\;=\;EVI_{obs}[doy]\;-\;EVI_{phen}[doy] \end{array}$$

where *EVI*_*obs*_ *[doy]* is the observed EVI at the day of the year within the year of the disturbance,

*EVI*_phen_ *[doy]* is the estimated EVI phenology at the day of the year of the reference period.

**Figure 6:**
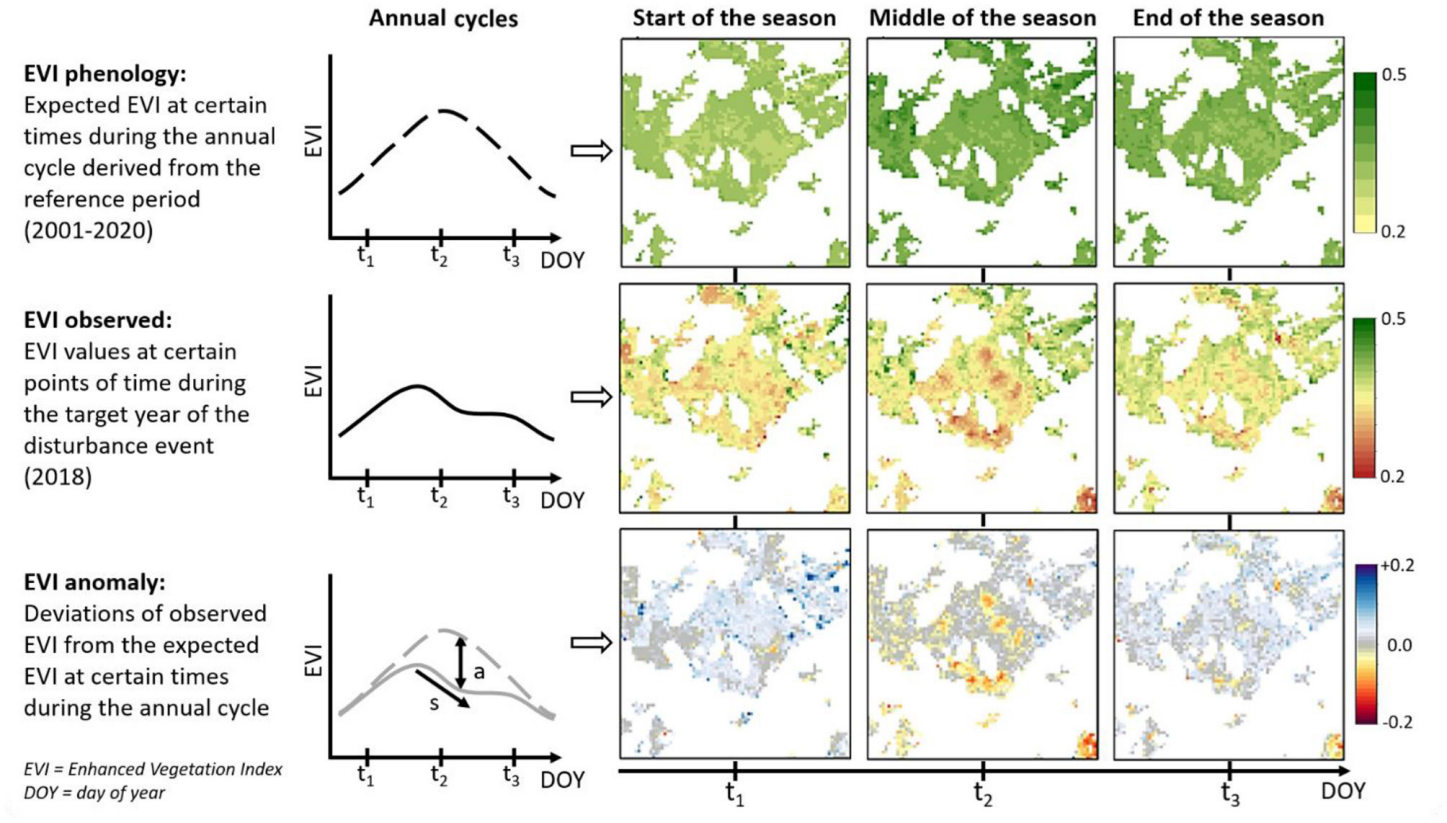
Theoretical framework of the EVI phenology (top) combining the phenological series of all investigated years for each individual pixel, thus being considered the specific baseline for each pixel. The observed EVI can be within the normal distribution or they can distinctly differ (middle). The difference between the EVI phenology curve and the observed curve is the anomaly (bottom). On the right is an example of the spatial distribution of the EVI from the start of the season to the end.

From each MODIS anomaly stack, the individual pixels, which passed the QA check, were extracted to provide the basis for the pattern analysis of the phenological time series (Figure 5, pixel extraction). The multi-temporal pixel stacks of known forest disturbances are used to derive prototypes of different disturbance patterns (Figure 5, pattern exploration). The disturbance is defined here by at least three consecutive values of negative deviation from the long-term phenological series, derived in step one, within the area covered by any individual pixel. We, therefore, gain one EVI phenology kernel density series for each individual pixel, which provides us with the expected EVI for this pixel over a year (Figure 6, top). The phenology is estimated based on kernel density estimation (KDE). A bivariate Gaussian kernel is centered around each individual observation, and the height of all kernels is averaged till the final density curve is established (Estay and Chávez, 2018). The KDEs define each anomaly value within the frequency distribution of the observed values at a given DOY. An anomaly is any value outside the 90% probability distribution of the referenced frequency distribution (RDF) (Decuyper et al., 2022). The curve is therefore specific to any pixel and provides a kind of fingerprint made up mainly of the specific species combination and the forest density, acting as a baseline. In the event of a known disturbance, the EVI curve differs from the expected EVI (Figure 6, middle). The difference between the observed EVI within a disturbance year and the expected EVI estimated from the long-term time series can be seen as the EVI anomaly (Figure 6, bottom).

Anomaly patterns were derived by scanning each multi-temporal pixel stack for their individual deviation from the long-term EVI phenology. The pixel-by-pixel analysis requires the screening of the individual pixel time series. The aim was to compare the observed EVI in the year of the potential disturbance event with the expected EVI, which is the long-term phenology estimated from the entire 20-year EVI time series at the individual pixel level. Due to this method, the variance in vegetation cover is lower than it would be expected if pixels were analyzed without being spatially explicit. It is important that all years are included to form the EVI_phen_ series, and therefore, no pre-selection is applied. A disturbance is detected when three consecutive events of the time series are identified by an anomaly. Using the metrics duration, amplitude, and slope, the different disturbance types can be identified. Using the pattern types, the causes can be further identified (Figure 1).

The phenology includes the green-up at the start of the vegetation period, reaching full foliation in summer, and the decline at the end of the season (Figure 6, right). This curve is specific to any pixel and is defined by the forest type, the species, and their mixture, as well as the understory modified by further abiotic factors. The EVI anomaly can be described by the duration (t_1_ to t_3_), the amplitude (a), and the slope (s). These parameters can be automatically extracted and describe the pattern of the anomaly. This was done by detecting a minimum of three consecutive and negative sequences (RDF ≥ 0.9) and calculating from these values the distance between the smallest value and the EVI phenology at this DOY.

## Results

The method provides an easy-to-use workflow for the distinction between abrupt and gradual disturbance types from the phenological series at a pixel level. Using the automatically extracted parameters of duration, amplitude, and slope, the derived patterns allow a further separation into some main causes: fire, storm, insect, and fungi (Figure 1) by pattern analysis.

### Distinct Change Types

**Figure 7:**
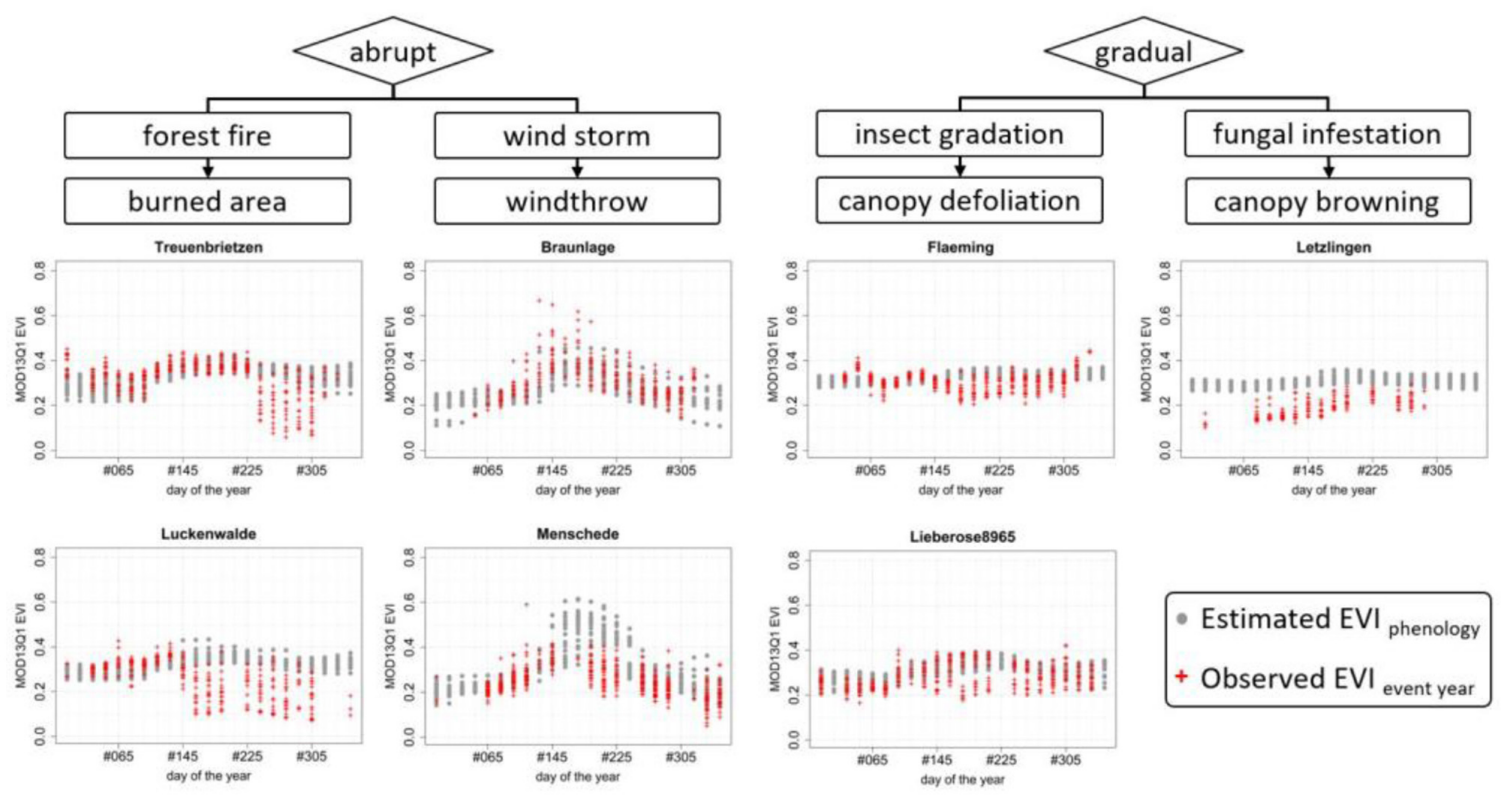
Visualization of abrupt and gradual change.

Enhanced Vegetation Index phenology provides an easy-to-track development of photosynthetic activity of the most upper forest canopy layer during the course of the year derived from the long-term reference series (20 years) and thus provides clear evidence of anomalous years. The simplest differentiation is between abrupt (e.g., fire, storm) and gradual (e.g., insects, fungi) disturbances (Figure 7), which are, in brief, identified by a sudden, negative deviation between the first and the third observation, or slow, when the decline last for more observations (slope) of the EVI_obs_ sequences compared to the EVI_phen_. In addition, the identification of fire damage is shown in a very abrupt decline of the EVI_obs_, and no recovery within the year of occurrence can be observed (Figure 7, first column). Windthrow, however, seems to be described by a sudden change which can be shown as an increase (location Braunlage) or decrease (location Menschede) (Figure 7, second column) of the EVI depending on the extent of the disturbed area and the prevalent forest type (deciduous vs. broadleaf). The identified patterns of disturbances are scattered and spatially unrelated (Figure 3; examples 3 and 4) which distinguished them from the linked and large-scale patterns of burned areas or large-scale clear cuts. Furthermore, the recovery in windthrow areas shows an increased EVI in the spring after the disturbance by the regeneration of the remaining understory or ground vegetation, which increases the EVI values. Gradual change by insect or fungal infestation is distinguished by a less pronounced decline (slope) and a smaller amplitude, at least at the beginning of the calamity and gradation period. While insects lead to slow defoliation of the canopy with a re-foliation possible in autumn (Figure 7, third column), fungi cause a canopy browning without recovery (Figure 7, fourth column). Gradual change therefore can be identified by a slow onset of the decline in the EVI and a remaining below the long-term mean for the entire vegetation period. The amplitude is generally smaller than with abrupt change. Between the two investigated cases of gradual change, canopy defoliation by insects occurred after DOY 145, whereas fungal infestations showed a continuous gradual decline from the beginning of the vegetation period onward over the entire year.

Selective tree harvest in line with the established forest management practice in Germany does not reach a detectable level provided by the MODIS (250 m by 250 m pixel size) due to the rapid closing of the canopy by the remaining trees. However, large-scale felling operations that exceed the individual pixel size, such as the TELSA Gigafactory construction site in Brandenburg, or sanitary felling in forest areas with extensive bark beetle infestation, can be detected and later verified using MODIS VI time series data (slope + amplitude).

### Magnitudes of Severity

In both, pixels with abrupt and those with gradual change, the assessment of the magnitude provides an indication of the severity of the disturbance and a differentiation between individual pixels. Here, the classification from the ground assessments relies on the consistent definition of thresholds, e.g., custom severity classification schemes. This, however, is easier for a finite event with spatial–temporally distinct impact, showing an abrupt decline in the EVI_obs_, compared to gradually evolving events without distinct temporal or spatial change markers.

**Figure 8:**
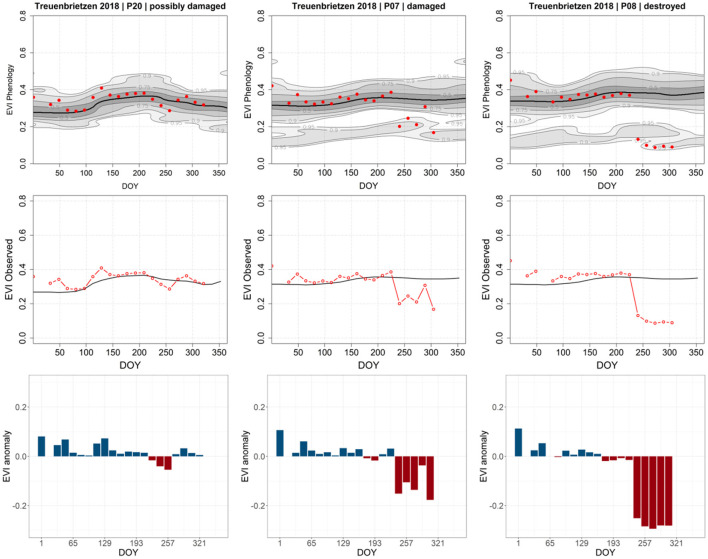
Annual EVI signatures for three different pixel locations grading from *possibly damaged* to *destroyed* (left to right): The EVI phenology (top) provides the baseline derived from the 20 investigated years, plus the year of the disturbance (red dots). The observed (black line, middle) is the center of the Gaussian kernel density, and the red line is the observed EVI in the year of the disturbance. The detected negative anomalies (bottom). The time and slope of the negative anomalies detected are coherent to the 2018 fire occurrence in Treuenbrietzen; the more the anomalous values approach the probability threshold (RDF ≥ 0.9), the greater the likeliness of a detected anomaly being severe.

Using the Copernicus emergency service grading map products as a reference in the spatial comparison, MODIS pixels across different burnt severity classes were selected from the MODIS EVI series. The overlay of the annual series of individual pixel compared to the long-term mean of those exact pixel provides a location-specific phenological series. The different extent of deviation in the event year, here 2018 (Figure 8), allows identification of an affected pixel and the spatial magnitude of the impact. The classification provides a quantitative interpretation of the meaning of the differenced normalized burn ratio (dNBR) results, and the term “severity” (Keeley, 2009) is therefore a qualitative term that could be quantified in different ways and is relative in regard to the most severe damage of the area or multiple areas.

The example of the forest fire in Treuenbrietzen (Figure 8) shows the potential of the method as individual pixels are assessed regarding their individual disturbance and its severity. This allows the detection of multiple disturbance causes in neighboring pixels.

**Figure 9:**
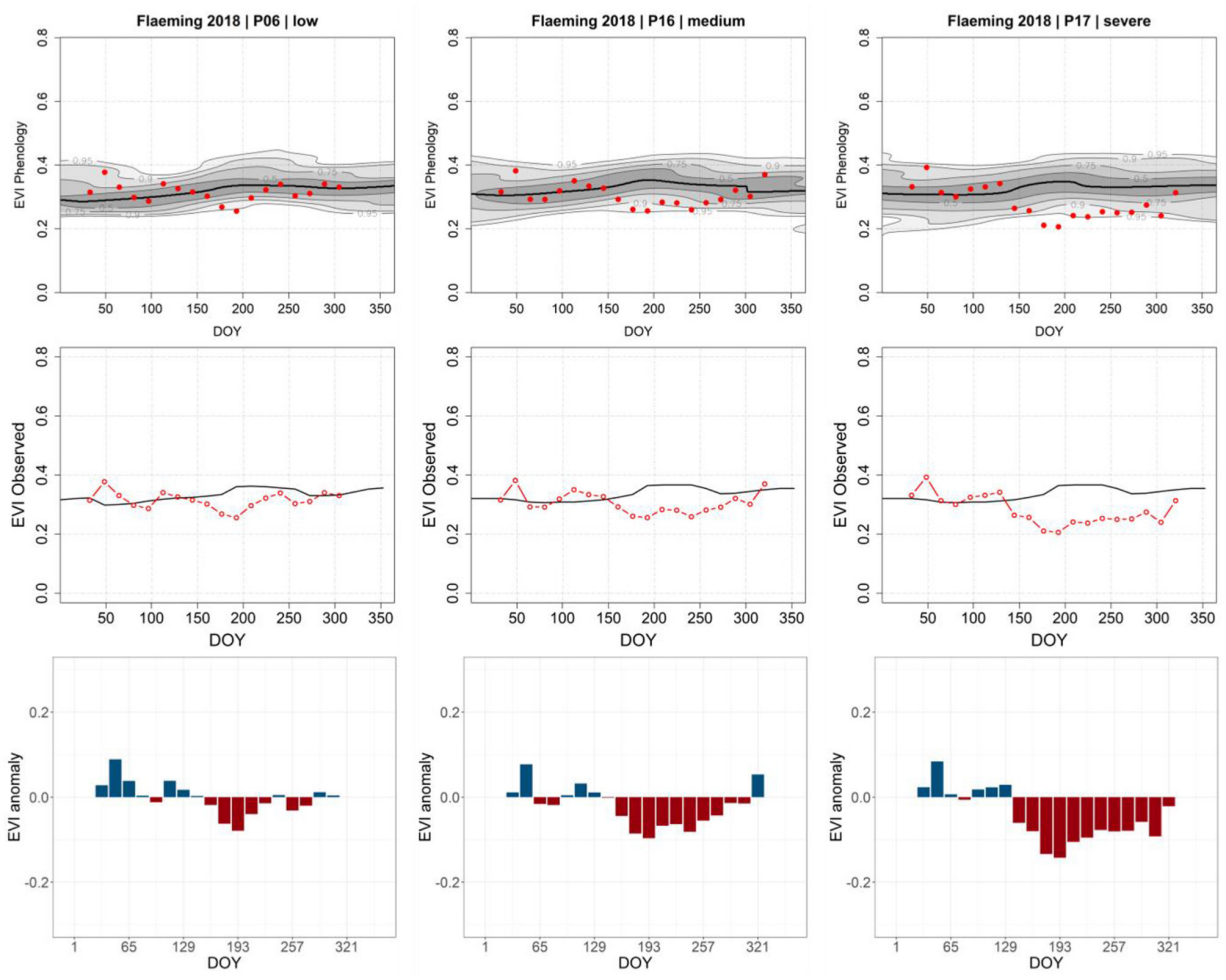
Annual EVI signatures for three different pixel locations grading from *low*, over *moderate*, to *severe defoliation* (left to right). The EVI phenology (top) provides the baseline derived from the 20 investigated years, plus the year of the disturbance (red dots). The observed (black line, middle) is the center of the Gaussian kernel density, the red line is the observed EVI in the year of the disturbance. The detected negative anomalies (bottom) correlate with the greening season and degree of defoliation due to the 2018 insect mass population in the Fläming region; the more the anomalous values approach the probability threshold (RDF ≥ 0.9), the greater the likeliness of a detected anomaly being severe disturbance interactions.

A similar approach was applied for pixels with insect defoliation but using the MODIS-based Forest Condition Index (FCI; https://un-spider.org/advisory-support/recommended-practices/recommended-practice-drought-monitoring/in-detail; 2021) for severity classification. The results show that, while the extracted patterns (Figure 9) are less pronounced than the patterns of the abrupt change in Figure 8, a determination of the magnitude of severity is possible. Extraction of time series for individual pixel and disturbance cause is expressed in a specific pattern of the EVI anomaly, which is defined by the slope and amplitude allowing the differentiation of moderately and severely disturbed stands.

**Figure 10:**
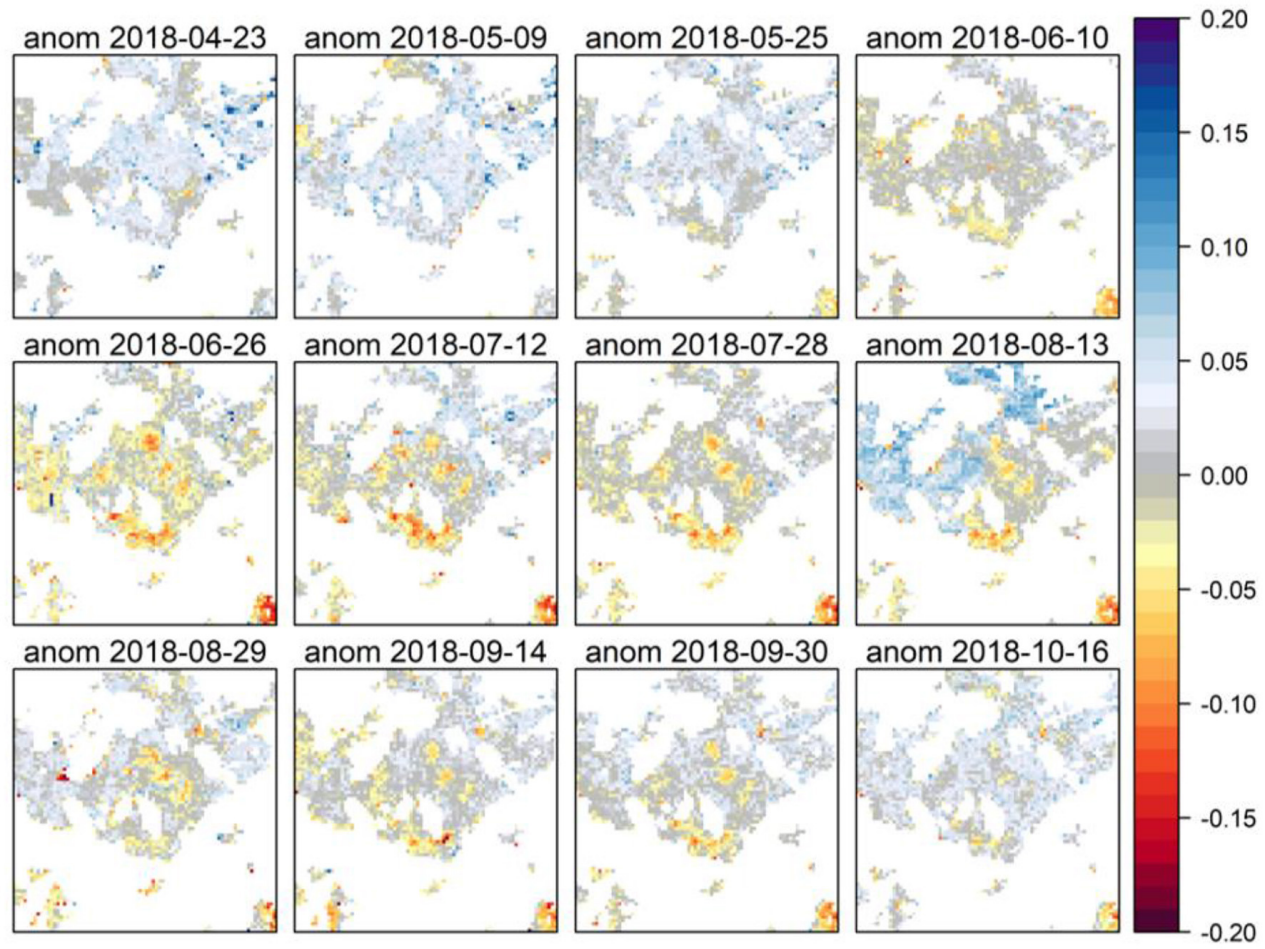
Detected EVI anomalies in the Fläming region (investigation site 6) during the vegetation period from the end of April to mid-October 2018. Red colors indicate a loss in EVI, blue colors a gain.

The comparison of the time series at the individual pixel level shows the clear differentiation at one site affected by the same insect-induced calamity during the same observation period. Therefore, this method allows the spatially explicit grading of the severity (Figure 10). The EVI series of the year 2018 shows a maximum greenness at the beginning of June, followed by clear signals of an EVI decline appears, marking the regional hotspots of insect defoliation. By mid of August, the picture changes again and some areas show signs of positive EVI anomalies; however, the major spatial hotspots of infestation remain a negative EVI anomaly in total till the end of the vegetation period.

**Figure 11:**
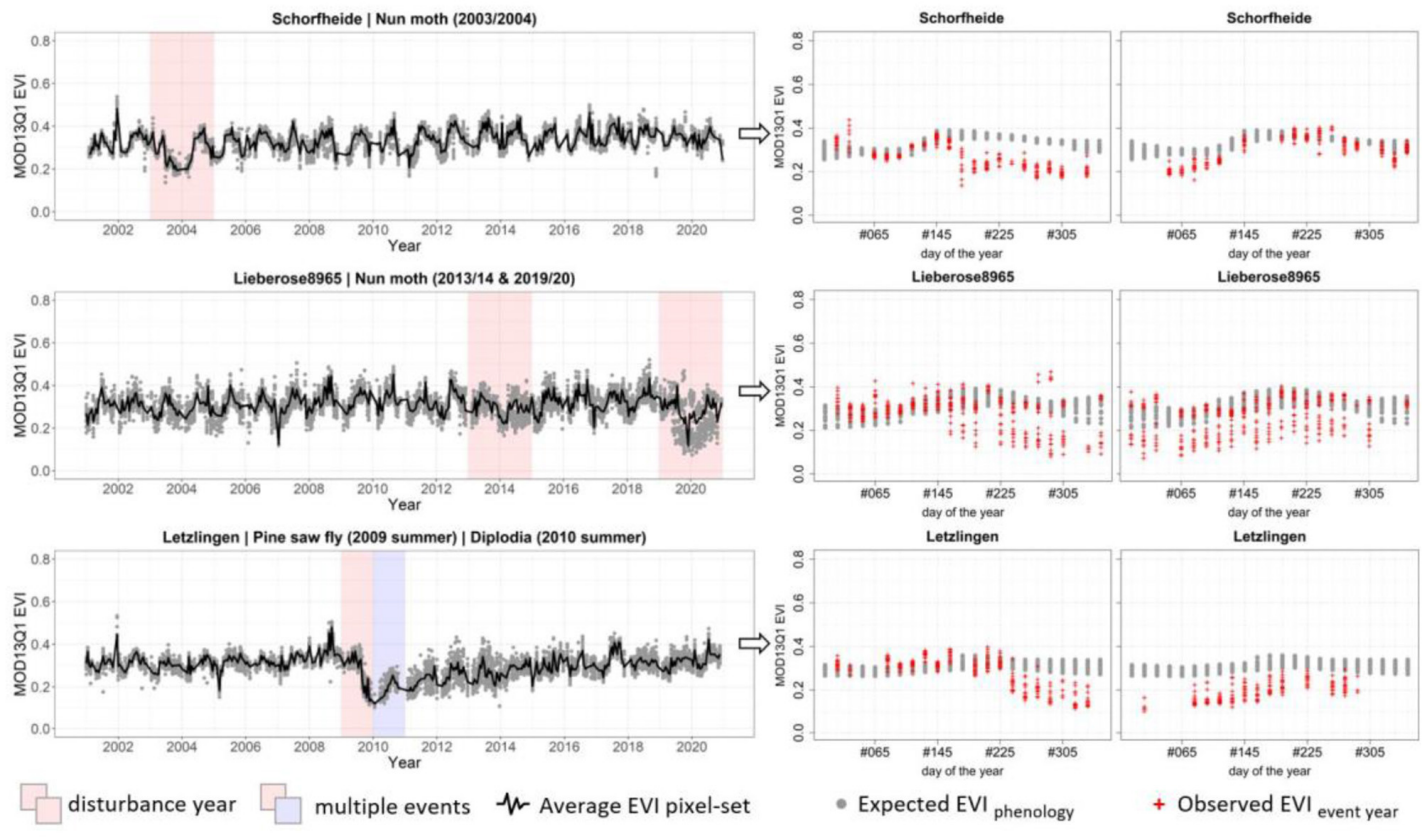
Comparison of gradual change patterns in 20-year time series signatures (left) detected at different time periods (indicated by colored boxes) and sampling sites with a diverse disturbance history and miscellaneous combinations of primary and secondary causing agents and the corresponding EVI sequences of the 2 consecutive years (right).

Using the 20 years of the phenological series, we were able to detect consecutive disturbance events coherent to the original set of ground assessed disturbances of insect calamities showing patterns of recovery at the beginning of the second greening season post-event (Figure 11, top); in addition to the reported 2-year disturbance episode, we spotted a second episode of likely repeated infestation (Figure 11, middle) as well as bi-annual disturbances caused by multiple events or different agents (Figure 11, bottom). This shows the potential of the method to include all years to get the phenological series for comparison, including the years with disturbances, and still detect those years as the patterns are distinctively different.

The analysis of phenological series compared to the assessment of individual time steps is less likely to cause false assessments. Due to the lack of precise tree species information and differences between regions in phenological series in timing as well as the different amounts of needle years supported by coniferous, single assessments can capture the time of needle shedding in 1 year and full needles in the second and therefore cause misinterpretation. Similar assessments can occur for broadleaves due to the various timing of leaf unfolding depending on the species.

**Figure 12:**
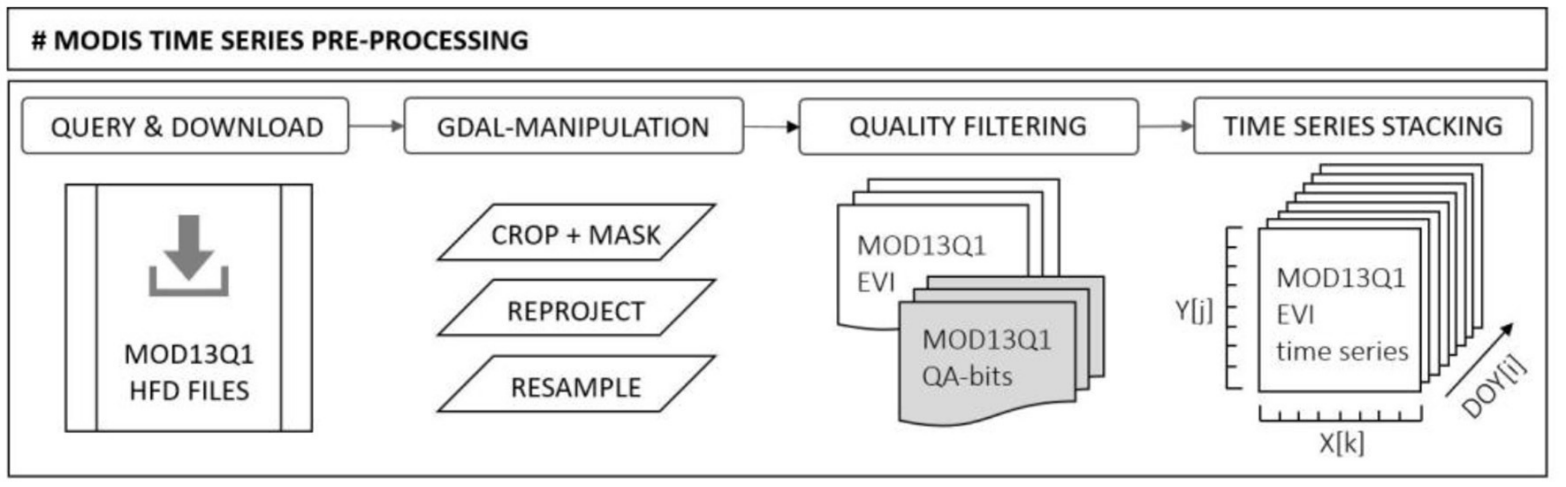
Workflow scheme of the MODIS pre-processing steps to obtain analysis-ready time-series image stacks.

## Discussion

Satellite change detection algorithms enable automated processing of large amounts of data, based on batch scripts, and thus can be used to establish monitoring routines that can serve as an early warning system. Change detection algorithms use deviations observed in VI signals of a certain observation period in relation to the “normal” long-term VI to detect the so-called phenological anomalies indicating a change in forest condition (Pasquarella et al., 2017). The schematic development of greenness during the vegetation period increases, without disturbance, to a maximum after which it is decreasing toward the end of the vegetation period. This development is interrupted by a disturbance event, causing a (significant) decrease in greenness prior to the phenological maximum indicating early defoliation (De Beurs and Townsend, 2008) or a more sudden decline after the maximum was reached. The accuracy of satellite-based forest change detection considerably depends on (a) the spectral, spatial, and temporal resolution of the time series data (Stenberg et al., 2008; Ghamisi et al., 2019), (b) the precise co-registration, standardization, and normalization of the multi-temporal images (Nguyen et al., 2020), and (c) the phenological estimation and change detection methods used (Atkinson and Urwin, 2012; Kandasamy et al., 2012; Petitjean et al., 2012). The common goal for disturbance detection is to use one method for any evaluation at a, at least, national scale. However, this leads to the multiple previously reported limitations which can roughly be classed in the lack of detection or overassessment, partly linked to the missing knowledge on individual species distribution, tempering the meaning within the analysis.

Thus, filling this gap regarding the current lack of operable methods mentioned by Atzberger et al., the method presented here can be used for all multispectral remote-sensing products providing a high intra-annual resolution and is expected to provide improved results as soon as possible, e.g., COPERNICUS mission provides sufficient years. Using the higher resolution of Sentinel or Landsat will benefit the spatial explicitly. Nevertheless, there are shortfalls. Atmospheric distortion variations in the background signal of observed pixels and clouds heavily affect obtained VI signatures (Pragnère et al., 1999), and thus, cloud masking is a crucial component in the pre-processing of optical satellite data for time series analysis.

While NDVI saturates over large water bodies and is prone to canopy background interactions in the presence of bare soil, red litter, or understory, EVI is expected to show a better performance where NDVI tends to mask the vegetation signal of the canopy due to the implemented background adjustment term (Huete et al., 1997). Regarding seasonality, the EVI values exhibited a smoother, more symmetrical seasonal profile with a narrower, well-defined peak greenness period. Furthermore, the EVI is very sensitive to needleleaf/broadleaf canopy structures with EVI values over needleleaf forests approximately one half of those over broadleaf forests resulting in sharper contrasts between the two forest types and a more pronounced broadleaf dry-down phase. Within the dynamic range, the EVI showed a smaller variation than that of the NDVI and a strong contrast in EVI values between broadleaf and needleleaf forests.

Using Sentinel 2A/B for establishing the phenological time series will, in 2022, still be less reliable due to the few available years. Due to the very high number and severity of disturbances linked to droughts, insects, fungi, fires, and storms, which occurred in the years 2017–2019, this will likely lead to a poorer detection rate of disturbances in consecutive years. Landsat does provide the longest time series at all, but it comes with the need for a high computing power and a lower temporal resolution, which might hamper the phenological series due to insufficient intra-annual scenes. Still, using MODIS data, the disturbed site needs to be above the pixel size, depending on the resolution of the remote-sensing product used, to be detectable. Ambiguous or indifferent phenomena occurring at the subpixel level caused by the individual decline of individual trees provide a mixed signal of the pixel. This problem is well known and linked to pixel resolution and the issue of mixed pixels (Schwalbe et al., 2006). The proposed pixel-by-pixel approach, therefore, remains at the level of the pixel resolution but does not consider their neighborhood relationships yet. This could, however, provide an important next step to describe the spatial extent and distribution of detected changes in the canopy of forests in terms of their uniformity and diversity, e.g., by means of the Shannon index (Spellerberg and Fedor, 2003), and thus further determine possible causes of disturbance in the respective study area.

Due to the lack of continuous long-term information on annual variation in the onset of the vegetation period (referring to the tree species-specific phenological phases as described in the ICP Forests documentation), additional expert knowledge and contextual information on the growth and weather conditions need to be implemented for adequate evaluation of the actual forest condition (Glenn et al., 2008). As concerned by Hansen et al. (2014), a miscomprehension of remote-sensing product validation is related to forest cover change in central Europe. Hence, detected forest loss can result from forest management in support of forest transition (forestry operation), due to insect infestation or water shortage defoliation (disturbance) or extensive removal due to bark beetle infestation (clearing operations). Whereas at the same time, potential loss of greenness in the most upper canopy layer might be masked by understory or shifts in the tree species composition after a storm, resulting in above-average greenness values due to seasonality effects introduced by mixed forests superimposing the long-term phenology signatures, which had been defined by needleleaf dominance in the pre-storm period.

An alternative approximation of the annual phenological baseline we have built on in this study is to use the observed frequency values and define the expected distribution directly from observed data without reference to a theoretical model (Chávez et al., 2019). The advantage of this approach is its flexibility to adapt to the particular conditions of every site and also to account for natural variability in annual phenology over time, which is smoothed over by the parametric functions. This approach is based on probabilistic estimations (Morio et al., 2015) of the annual phenology, from which disturbances measured as anomalies from the expected phenology can be assessed in terms of the frequency distribution of historical records, providing both a map showing the likelihood of the change detection and the change detection result itself. Current methods based on the parametric functions lack such a likelihood measure (Berger and Wolpert, 1984).

Other inaccuracies such as the spatial uncertainties of remote-sensing products remain relatively low in both daily and composited products (Huete et al., 2002) when using MODIS VI products for this study the geolocation accuracy (co-registration) allowing a pixel-to-pixel comparison through time was especially important which is given by MODIS data to subpixel accuracy, approaching the operational MODIS geolocation goal of 50 m (1r) at nadir (Wolfe et al., 2002).

Therefore, the advantages are that (a) 2-dimensional KDE (bi-variate Gaussian kernel) can capture any functional form, (b) the same number of points per cycle is not mandatory, and (c) the magnitude of the anomaly can be leveled at a probabilistic base.

Atmospheric distortions and variations in the background signal of observed pixels heavily affect obtained VI signatures (Pragnère et al., 1999), and thus, cloud masking is a crucial component in the pre-processing of optical satellite data for time series analysis. The pixel-by-pixel time series approach provides a flexible method, which is transferable to various vegetation cover types and conditions and is reliable across phenology regimes and disturbances. The non-parametric phenology estimation and kernel density-based change detection algorithms are robust to missing data and noise, which are especially related to the distinction of anomalies from noise (e.g., due to clouds, geometric errors) and time series reconstruction is independent of curve-fitting models for smoothing and generalization and change detection does not rely on artificially set thresholds. Therefore, the change detection result does not rely on user defined threshold (=artificially introduced thresholds are a source of uncertainty) (a) 2-dimensional KDE (Gaussian kernel) can capture any functional form, (b) the same number of points per cycle is not mandatory, and (c) the magnitude of the anomaly can be judged at a probabilistic base.

Forest ecosystems move through different phases, from seedlings to mature trees to the death of individuals or stands. Therefore, there was always a previous disturbance (Jentsch and White, 2019), and there will always be one following (Holling, 1973). Disturbances alter the structure and composition, and they influence their neighbors and shape large-scale patterns (Hart and Kleinman, 2018). However, in recent times, with large-scale forest disturbances and decline, the need for a fast and reliable assessment arises to extract disturbance patterns and causes (Vaglio Laurin et al., 2021), thus enabling aid recovery processes. Therefore, remote sensing provides a valuable addition to understanding forest disturbances but still needs ground-truthing, reliable monitoring data, and expert knowledge. To do so, the use of the EVI phenological series proved valuable. Specifically, the pixel-by-pixel approach is independent of a prior species determination, which is still problematic to do reliably with remote sensing, thus reducing the risk of false alarms due to the timing of the assessment within the phenological series, as well as the assuming the wrong species. A step-wise approach classifying first abrupt vs. gradual change to later gain more details but adding further information, whether by ground-truthing or using different bands, further reduces the overestimation of disturbances.

## Conclusion

In this study, we used a combination of ground assessments and spatially explicit EVI phenological time series to develop an identification scheme for four different forest disturbances. The patterns identified and validated by the ground assessments reliably showed enough further disturbance events. This method can distinguish abrupt and gradual changes in forests using robust remote-sensing products while a combination of ground assessments and remote-sensing time series is still needed for accuracy assessment and a precise determination of the type of insect or fungi.

Remote-sensing techniques are widely used for forest monitoring applications. Due to the complexity of the cause-and-effect relationships of forest ecosystems and the variety of factors involved, the stress-response of forests and trees has not been fully decoded yet. In the context of climate change, an in-depth understanding of this relationship is crucial for forest transformation and adaptation (Keenan, 2015; White et al., 2016). Thus, the differentiation and quantification of factors and their contribution to forest degradation and disturbance require further research. While we believe that remote sensing is an important addition to ground-based monitoring systems such as ICP Forests, we believe that methods need to be harmonized and validated in a similar way to provide valid information.

## Data Availability Statement

The datasets presented in this study can be found in online repositories. The names of the repository/repositories and accession number(s) can be found below: Kyrill windthrow data by WaldInfo.NRW | OpenGeodata 2021, under dl-de/by-2-0: https://www.opengeodata.nrw.de/produkte/umwelt_klima/wald_forst/wald/windwurfschadflaechen-kyrill_EPSG25832_Shape.zip; Copernicus Emergency Management Service © 2019 European Union, [EMSR266] Bad Karlshafen: Delineation Map, v1: https://emergency.Copernicus.eu/mapping/list-of-components/EMSR266; Copernicus Emergency Management Service © 2018 European Union, [EMSR307] Jüterbog: Delineation Map, Monitoring 1: https://emergency.Copernicus.eu/mapping/list-of-components/EMSR307; Copernicus Emergency Management Service © 2019 European Union, [EMSR363] Luckenwalde: Delineation Map, Monitoring 3: https://emergency.Copernicus.eu/mapping/list-of-components/EMSR363; Forstliche Versuchsflächen des Landesbetriebes Forst Brandenburg: (Silvicultural monitoring area for nun moth (Lymantria monacha) defoliation and Metadata: Forstliche Versuchsflächen des Landes Brandenburg Forstliche Umweltkontrolle Geodatensatz https://www.metaver.de/trefferanzeige?cmd=doShowDocument&docuuid=CAEA58C3-EA6D-4BCC-B995-A48CDDD3A0FC&plugid=/ingrid-group:ige-iplug-bb (accessed: May 09, 2022); Resource Reviere/Forstämter Sachsenanhalt https://www.waldgeoportal.de/layers/lzw_geonode_data:geonode:Waldflaeche_FGK (accessed: May 09, 2022); disturbance meta data “Pilotstudie zu den lokalen Auswirkungen des Klimawandels auf die Forstwirtschaft in ausgewählten Regionen Sachsen-Anhalts”; LZW Betr.-FA Letzlingen, Rev. Wannefeld: https://www.nw-fva.de/fileadmin/user_upload/Verwaltung/Publikationen/2015/NWFVA_Bd13_2015_Klimawandel.pdf; Insektenfraß-Schäden / Monitoring BB: https://forst.brandenburg.de/sixcms/media.php/9/bwin_verlust.pdf; https://forst.brandenburg.de/sixcms/media.php/9/efs62.pdf; https://www.researchgate.net/publication/236960911_Untersuchung_zur_Quantifizierung_von_Befallsflachen_phytophager_Insekten_in_Kiefern-Bestanden_mit_Hilfe_von_Fernerkundungs-Methoden_am_Beispiel_des_Nonnenraupenfrasses_im_Jahre_2003_in_der_Schorfheide; Treuenbritzen Waldbrand – Detail _&gt; “Die Auswirkungen des Dürre-jahres 2018 auf den Wald in Brandenburg”: https://forst.brandenburg.de/sixcms/media.php/9/efs67.pdf Waldzustandsbericht Brandenburg 2018; https://mluk.brandenburg.de/cms/media.php/lbm1.a.3310.de/Waldzustandsbericht_BB_2018.pdf p. 22.

## Author Contributions

AG and TS jointly developed the idea and developed the methods. AG contributed to data acquisition, preparation, and processing, as well as preparation of the manuscript. TS initiated the study, writing, literature research, and final version of the manuscript. Both authors contributed to the article and approved the submitted version.

## Funding

AG was supported by funding from the FNR in Germany within the project ErWiN—Grant Number: 2219WK54A.

## Author Disclaimer

All data used in this study are free and publicly available. The programs used are open-source applications and can be run on a standard computer, independent of the operating system and across platforms. The individual data processing routines are designed to be resource-efficient and make no special demands on memory or computer performance. Processing results can be stored locally and exported in various standard formats (e.g. GeoTiff, csv) and are compatible with common GIS applications. The proposed batch script functionality allows existing time series to be automatically scheduled or manually updated at any time with newly available satellite data, making it a useful satellite-based monitoring tool and early warning system to complement terrestrial surveys and 660 forest health surveys in operational forestry (see Figure 12).

## Conflict of Interest

The authors declare that the research was conducted in the absence of any commercial or financial relationships that could be construed as a potential conflict of interest.

## Publisher's Note

All claims expressed in this article are solely those of the authors and do not necessarily represent those of their affiliated organizations, or those of the publisher, the editors and the reviewers. Any product that may be evaluated in this article, or claim that may be made by its manufacturer, is not guaranteed or endorsed by the publisher.
