# Supplementary material for: Distinguishing Abrupt and Gradual Forest Disturbances With MODIS-Based Phenological Anomaly Series
Source: Front Plant Sci. 2022 May 23;13:863116. doi: 10.3389/fpls.2022.863116 (PMC9168887; doi:10.3389/fpls.2022.863116)
Supplement: Supplementary file 1 [file Data_Sheet_1.PDF]

Supplement 1

List of German forest condition monitoring approaches

- https://forestwatch.lup-umwelt.de/app/
- http://waldzustandsmonitor.de/interaktiv/
- https://map3d.remote-sensing-solutions.de/waldmonitor-deutschland/#
- Ibisch et al Für Greenpeace
- https://www.dlr.de/eoc/desktopdefault.aspx/tabid-17417/27631_read-73228/
- https://www.ufz.de/index.php?de=48150
