# Supplementary material for: Distinguishing Abrupt and Gradual Forest Disturbances With MODIS-Based Phenological Anomaly Series
Source: Front Plant Sci. 2022 May 23;13:863116. doi: 10.3389/fpls.2022.863116 (PMC9168887; doi:10.3389/fpls.2022.863116)
Supplement: Supplementary file 2 [file Data_Sheet_2.PDF]

SUPPLEMENT 2

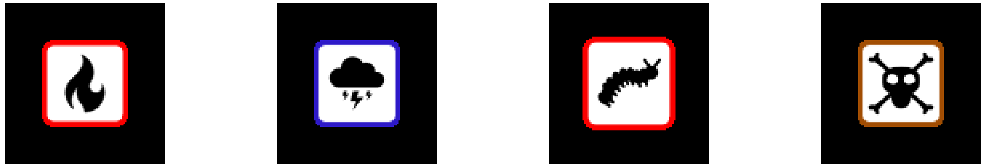

SATELLITE DATA
REFERENCES

Global Forest Change 2000–2020 Data Download

https://data.globalforestwatch.org/documents/134f92e59f344549947a3eade9d80783/explore | License and credits: Source: Hansen/UMD/Google/USGS/NASA

Citation:

Hansen, M. C., P. V. Potapov, R. Moore, M. Hancher, S. A. Turubanova, A. Tyukavina, D. Thau, S. V. Stehman, S. J. Goetz, T. R. Loveland, A. Kommareddy, A. Egorov, L. Chini, C. O. Justice, and J. R. G. Townshend. 2013. "High-Resolution Global Maps of 21st-Century Forest Cover Change." Science 342 (15 November): 850–53. Data available on-line from: http://earthenginepartners.appspot.com/science-2013-global-forest.

Dataset Details:

This global dataset is divided into 10x10 degree tiles, consisting of seven files per tile. All files contain unsigned 8-bit values and have a spatial resolution of 1 arc-second per pixel, or approximately 30 meters per pixel at the equator.

Tree canopy cover for year 2000 (treecover2000): Tree cover in the year 2000, defined as canopy closure for all vegetation taller than 5m in height. Encoded as a percentage per output grid cell, in the range 0–100.
Global forest cover gain 2000–2012 (gain): Forest gain during the period 2000–2012, defined as the inverse of loss, or a non-forest to forest change entirely within the study period. Encoded as either 1 (gain) or 0 (no gain).
Year of gross forest cover loss event (lossyear): Forest loss during the period 2000–2020, defined as a stand-replacement disturbance, or a change from a forest to non-forest state. Encoded as either 0 (no loss) or else a value in the range 1–20, representing loss detected primarily in the year 2001–2020, respectively.
Data mask (datamask): Three values representing areas of no data (0), mapped land surface (1), and permanent water bodies (2).
Circa year 2000 Landsat 7 cloud-free image composite (first): Reference multispectral imagery from the first available year, typically 2000. If no cloud-free observations were available for year 2000, imagery was taken from the closest year with cloud-free data, within the range 1999–2012.
Circa year 2020 Landsat cloud-free image composite (last): Reference multispectral imagery from the last available year, typically 2020. If no cloud-free observations were available for year 2020, imagery was taken from the closest year with cloud-free data.

Reference composite imagery are median observations from a set of quality assessed growing season observations in four spectral bands, specifically Landsat bands 3, 4, 5, and 7. Normalized top-of-atmosphere (TOA) reflectance values (ρ) have been scaled to an 8-bit data range using a scale factor (g): $DN = \rho \cdot g + 1$

The g factor was chosen independently for each band to preserve the band-specific dynamic range, as shown in the following table:

Landsat Band	g
Band 3 (red)	508
Band 4 (NIR)	254
Band 5 (SWIR)	363
Band 7 (SWIR)	423

MODIS/Terra Vegetation Continuous Fields Yearly L3 Global 250m SIN Grid (MOD44B)

Product info: https://lpdaac.usgs.gov/products/mod44bv006/
User guide: https://lpdaac.usgs.gov/documents/112/MOD44B_User_Guide_V6.pdf
https://ladsweb.modaps.eosdis.nasa.gov/missions-and-measurements/products/MOD44B#product-information

**Shortname:**	MOD44B
**Platform:**	Terra
**Instrument:**	MODIS
**Processing Level:**	Level-3
**Spatial Resolution:**	250 m
**Temporal Resolution:**	annual
**ArchiveSets:**	6
**Collection:**	MODIS Collection 6 (ArchiveSet 6)
**PGE Number:**	PGE61
**File Naming Convention:**	MOD44B.AYYYYDDD.hHHvVV.CCC.YYYYDDDHHMMSS.hdf<br>•YYYYDDD = Year and Day of Year of acquisition<br>•hHH = Horizontal tile number (0-35)<br>•vVV = Vertical tile number (0-17)<br>•CCC = Collection number<br>•YYYYDDDHHMMSS = Production Date and Time
**Citation:**	John Townsend, Charlene DiMiceli - University of Maryland and MODAPS SIPS - NASA. (2015). MOD44B MODIS/Terra Vegetation Continuous Fields Yearly L3 Global 500m SIN Grid. NASA LP DAAC. http://doi.org/10.5067/MODIS/MOD44B.006
**Keywords:**	Climate Change, Canopy Characteristics, Biomass, Land Cover

MODIS/ Terra + Aqua Land Cover Type Yearly L3 Global 500m SIN Grid (MCD12Q1)

Product info: https://lpdaac.usgs.gov/products/mcd12q1v006/
User guide: https://lpdaac.usgs.gov/documents/101/MCD12_User_Guide_V6.pdf
https://ladsweb.modaps.eosdis.nasa.gov/missions-and-measurements/products/MCD12Q1#product-information

Shortname:	MCD12Q1
Platform:	Combined Aqua Terra
Instrument:	MODIS
Processing Level:	Level-3
Spatial Resolution:	500 m
Temporal Resolution:	annual
ArchiveSets:	6
Collection:	MODIS Collection 6 (ArchiveSet 6)
PGE Number:	PGE41
File Naming Convention:	MCD12Q1.AYYYYDDD.hHHvVV.CCC.YYYYDDDHHMMSS.hdf<br>•AAYYYYDDD = Acquisition Year and Day of Year<br>•hHH = Horizontal tile number (0-35)<br>•vVV = Vertical tile number (0-17)<br>•CCC = Collection number<br>•YYYYDDDHHMMSS = Production Date and Time
Citation:	Mark Friedl, Damien Sulla-Menashe - Boston University and MODAPS SIPS - NASA. (2015). MCD12Q1 MODIS/Terra+Aqua Land Cover Type Yearly L3 Global 500m SIN Grid. NASA LP DAAC. http://doi.org/10.5067/MODIS/MCD12Q1.006
Keywords:	Climate Change, Climate Modeling, Land Cover

SUPPLEMENT

Supplement X:

USING EVI PATTERNS OF SATELLITE TIME SERIES TO DETECT FOREST PHENOLOGY ANOMALIES

Study sites				MCD12Q1 Land Cover Type				Disturbance reference polygons					Pixel samples			
AOI	study site locality	event date	observed event	Forest cover [%]	Evergreen Needleleaf [%]	Deciduous Broadleaf [%]	Mixed Forest [%]	polygon count [N]	sum area [ha]	avg. area [ha]	min. area [ha]	max. area [ha]	Pixel sample [N]	Needle Forest	Mixed Forest	Other Cover
1	NSG Hemer	2007-01-18	Storm Kyrill	50.5	12.4	0.0	87.6	1145	4273.6	3.7	0.0	211.6	40	20	20	0
2	Menschede	2007-01-18	Storm Kyrill	61.6	22.6	0.0	77.4	1114	3682.0	3.3	0.0	187.3	40	20	19	1
3	Bad Karlshafen	2018-01-18	Storm Friederike	39.0	0.2	19.9	80.0	554	862.7	1.6	0.0	28.9	20	0	18	2
4	Braunlage	2018-01-18	Storm Friederike	96.8	58.4	2.2	39.4	207	344.4	1.7	0.1	20.2	20	20	0	0
5	Letzlingen	2009/10	Diplodia	37.8	86.4	4.8	8.8	21	403.8	19.2	2.2	223.4	20	20	0	0
6	Flaeming	2018	Nun moth	50.9	93.5	0.2	6.3	77	1550.3	20.1	0.5	648.9	20	20	0	0
7a	Treuenbrietzen	2018-08-23	Forest fire	28.9	85.7	4.3	10.0	1	656.1	656.1	656.1	656.1	20	20	0	0
7b	Luckenwalde	2019-06-03	Forest fire	28.2	87.8	3.1	9.1	4	556.0	139.0	8.1	283.7	20	18	0	2
8	Schorfheide	2003/04	Nun moth	73.2	58.2	0.6	41.2	31	192.5	6.2	2.1	29.8	20	20	0	0
9	Lieberose	2013/14	Nun moth	49.5	94.2	0.5	5.3	510	1084.3	2.1	0.3	55.2	20	20	0	0

Supplement X: Forest Condition Index

Forest Condition Index (FCI)

$$CI_{ijk} = \sum \frac{(EVI_{obs} - EVI_{pred})}{RMSE} / n_{obs}$$

Where:

CI_{ijk}	Condition for the pixel i at the time j for the year k (observation period 2018);
EVI_{obs}	Observed EVI for the pixel i at the time j for the year k (observation period 2018);
EVI_{pred}	Predicted EVI for the pixel i at the time j period within the years 2009 - 2017;
n_{obs}	Number of valid values at each pixel at the time j within the years 2009 – 2017

Root Mean Square Error (RMSE)

$$RMSE = \sqrt{\sum_{i=1}^{n} \frac{(\hat{y}_i - y_i)^2}{n}}$$

Where:

$\hat{y}_1, \hat{y}_2, \ldots, \hat{y}_n$ are predicted EVI values (phenology)

$y_1, y_2, \ldots, y_n$ are observed EVI values

n is the number of observations

V. Pasquarella, B. Bradley, and C. Woodcock, 'Near-Real-Time Monitoring of Insect Defoliation Using Landsat Time Series', Forests, vol. 8, no. 8, p. 275, Jul. 2017, doi: 10.3390/f8080275. https://www.mdpi.com/1999-4907/8/8/275

Supplement X:
USING EVI PATTERNS OF SATELLITE TIME SERIES TO DETECT FOREST PHENOLOGY ANOMALIES

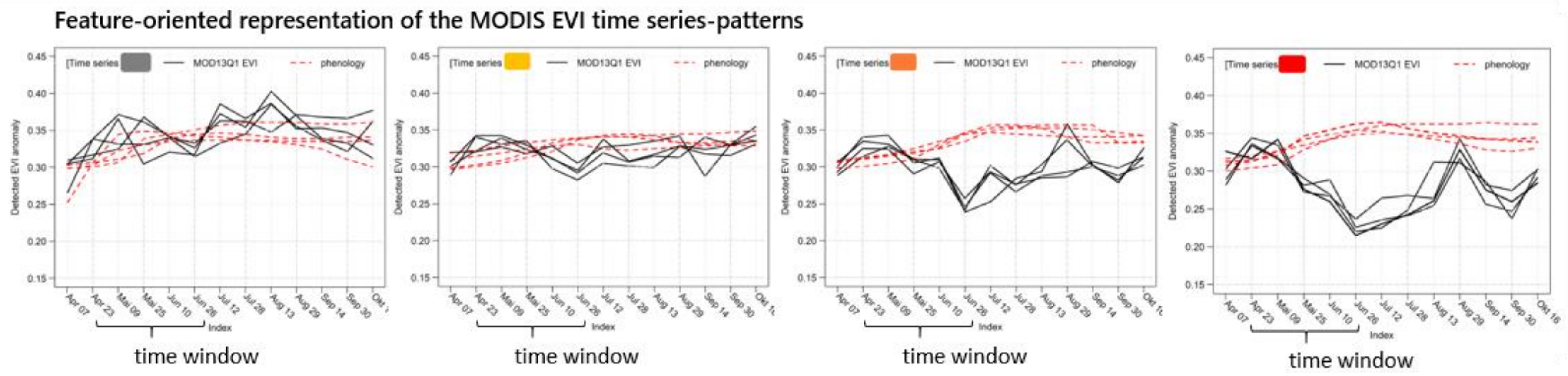

(A) forest disturbance map

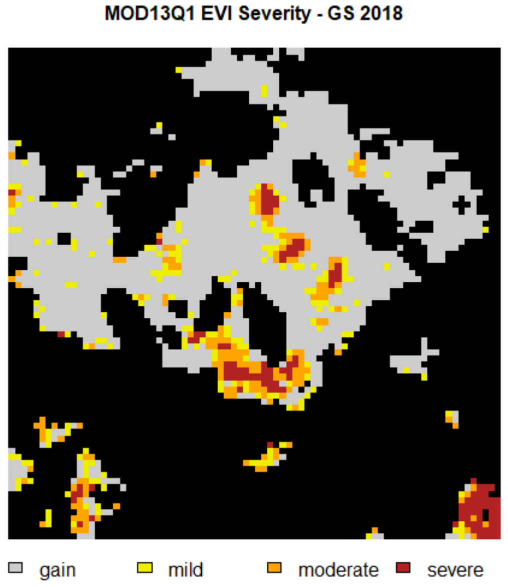

(B) Severity assessment

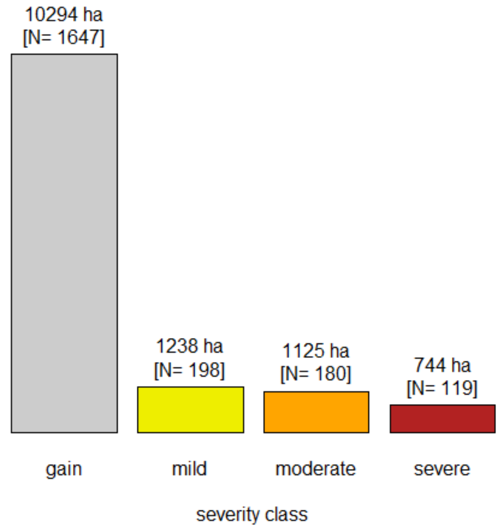

(C) Anomaly pattern extraction

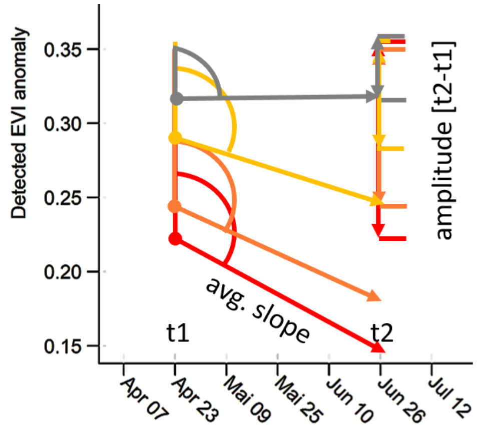

Source: M.Sc. Thesis A. Gnilke HNEE FIT 2020; "Using clustered EVI patterns of satellite time series to detect forest phenology anomalies – A multi-scale approach to assess insect defoliation in pine forests"

Supplement X:

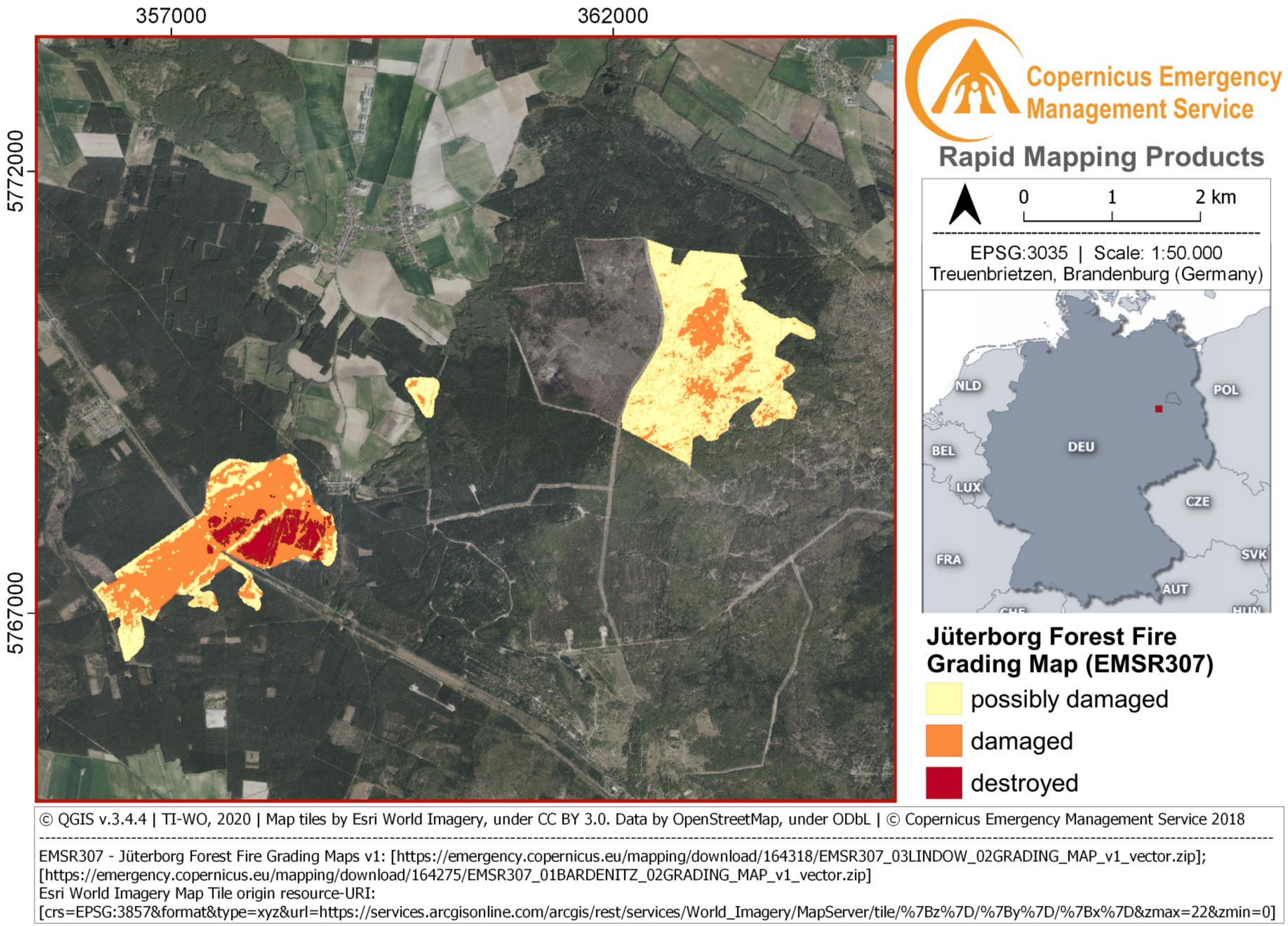

SUPPLEMENT X:
MODIStsp image pre-processing

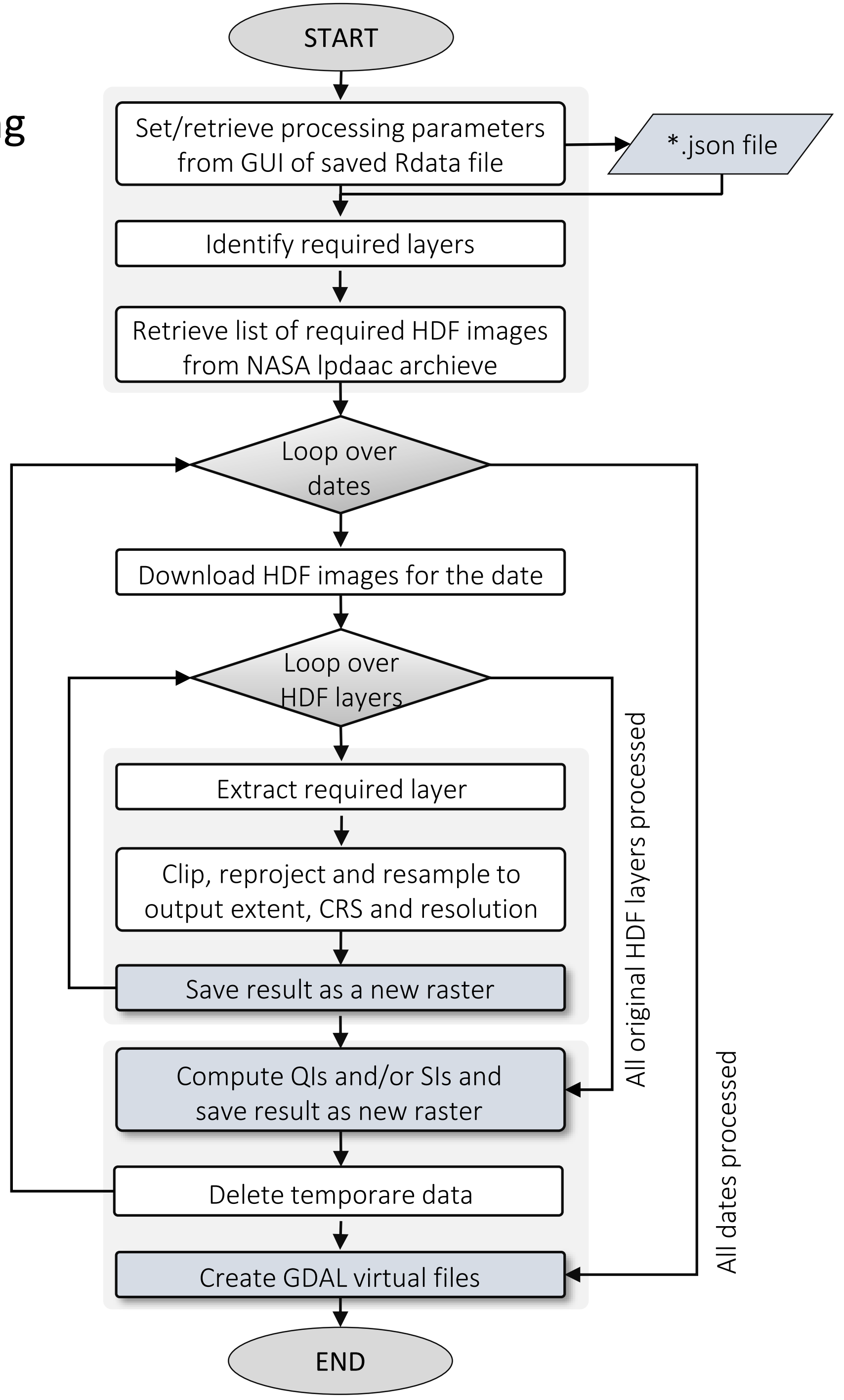

Search criteria and processing parameters

Selected sensor:	Terra, MODIS
Product version:	v006
List of products:	MOD13Q1 VI 16-Day L3
Selected bands:	[1,1,1,0,0,0,0,1,0,0,0,1]
Period from:	2009/01/01
Period to:	2018/10/31
Bounding box min:	X: 342910, Y: 5778780
Bounding box max:	X: 363910, Y: 5797780
Selected tiles:	h18v03
Target CRS:	EPSG 32633
Qualityband sel:	[1,1,1,1,1,1,1,1,1]
Spatial resolution:	250m (resampled)
Resampling method:	nearest neighbor
Output format:	Gtiff - INT16

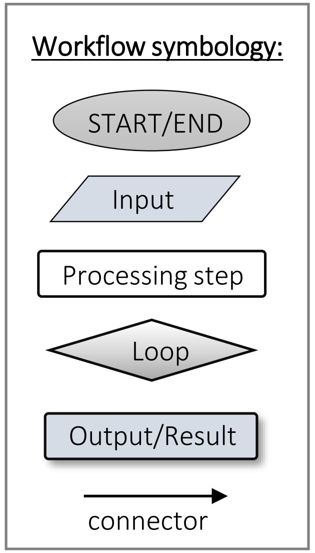

SUPPLEMENT X: MODIS quality filtering

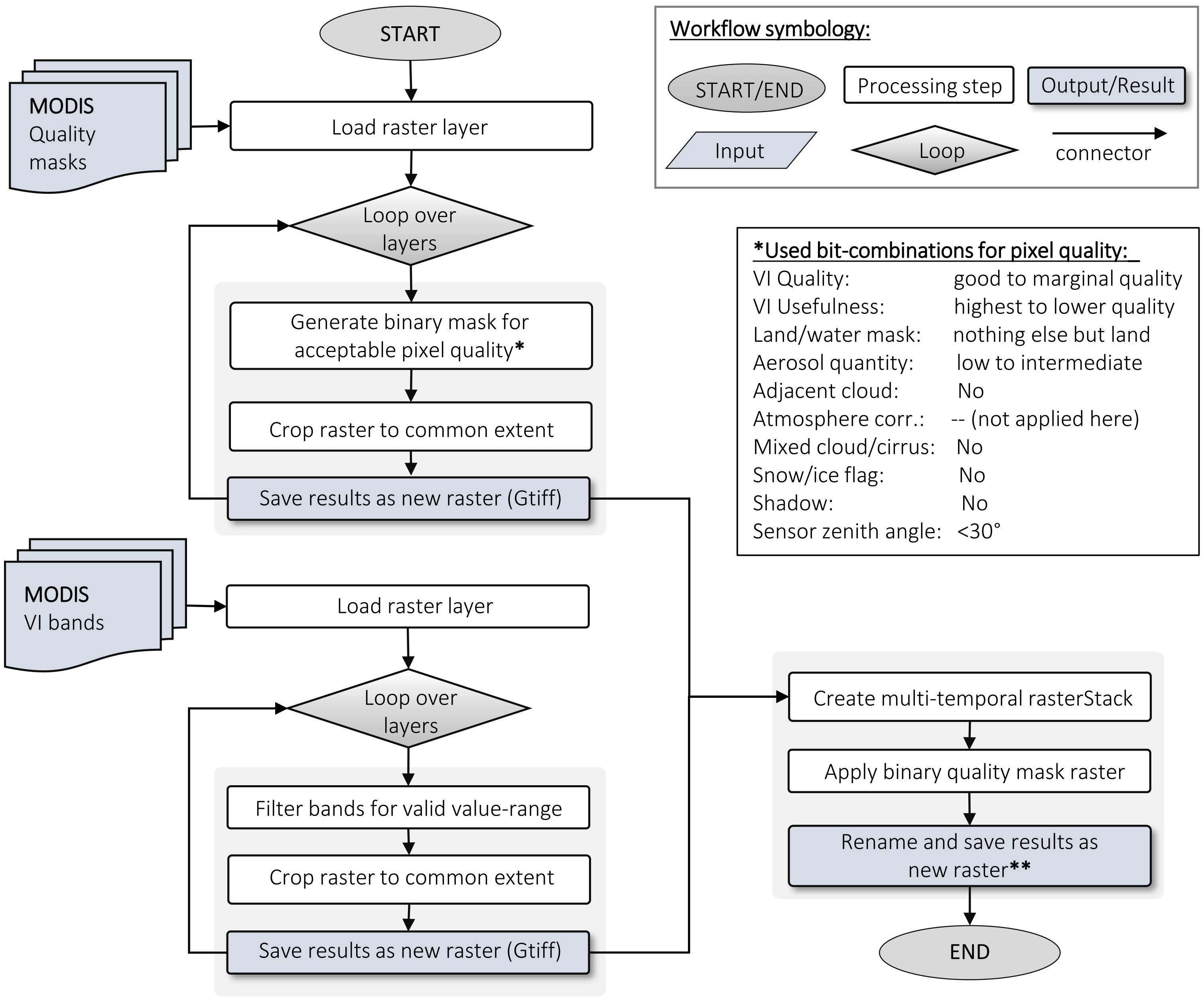

***Used bit-combinations for pixel quality:**

VI Quality:	good to marginal quality
VI Usefulness:	highest to lower quality
Land/water mask:	nothing else but land
Aerosol quantity:	low to intermediate
Adjacent cloud:	No
Atmosphere corr.:	-- (not applied here)
Mixed cloud/cirrus:	No
Snow/ice flag:	No
Shadow:	No
Sensor zenith angle:	<30°

Bits	ParameterName	Value	Description
0-1	VI Quality (MODLAND QA Bits)	**00**	**VI produced with good quality**
		**01**	**VI produced, but check other QA**
		10	Pixel produced, but most probably cloudy
		11	Pixel not produced due to other reasons
2-5	VI Usefulness	**0000**	**Highest quality**
		**0001**	**Lower quality**
		0010	Decreasing quality
		0100	Decreasing quality
		1000	Decreasing quality
		1001	Decreasing quality
		1010	Decreasing quality
		1100	Lowest quality
		1101	Quality so low that it is not useful
		1110	L1B data faulty
		1111	Not useful/not processed
6-7	Aerosol Quantity	**00**	**Climatology**
		**01**	**Low**
		**10**	**Intermediate**
		11	High
8	Adjacent cloud detected	**0**	**No**
		1	Yes
9	Atmosphere BRDF Correction*	0	No
		1	Yes
10	Mixed Clouds	0	No
		1	Yes
11-13	Land/Water Mask	000	Shallow ocean
		**001**	**Land (Nothing else but land)**
		010	Ocean coastlines and lake shorelines
		011	Shallow inland water
		100	Ephemeral water
		101	Deep inland water
		110	Moderate or continental ocean
		111	Deep ocean
14	Possible snow/ice	**0**	**No**
		1	Yes
15	Possible shadow	**0**	**No**
		1	Yes

* not implemented for this area

Fläming summer 2018
Nun moth defoliation

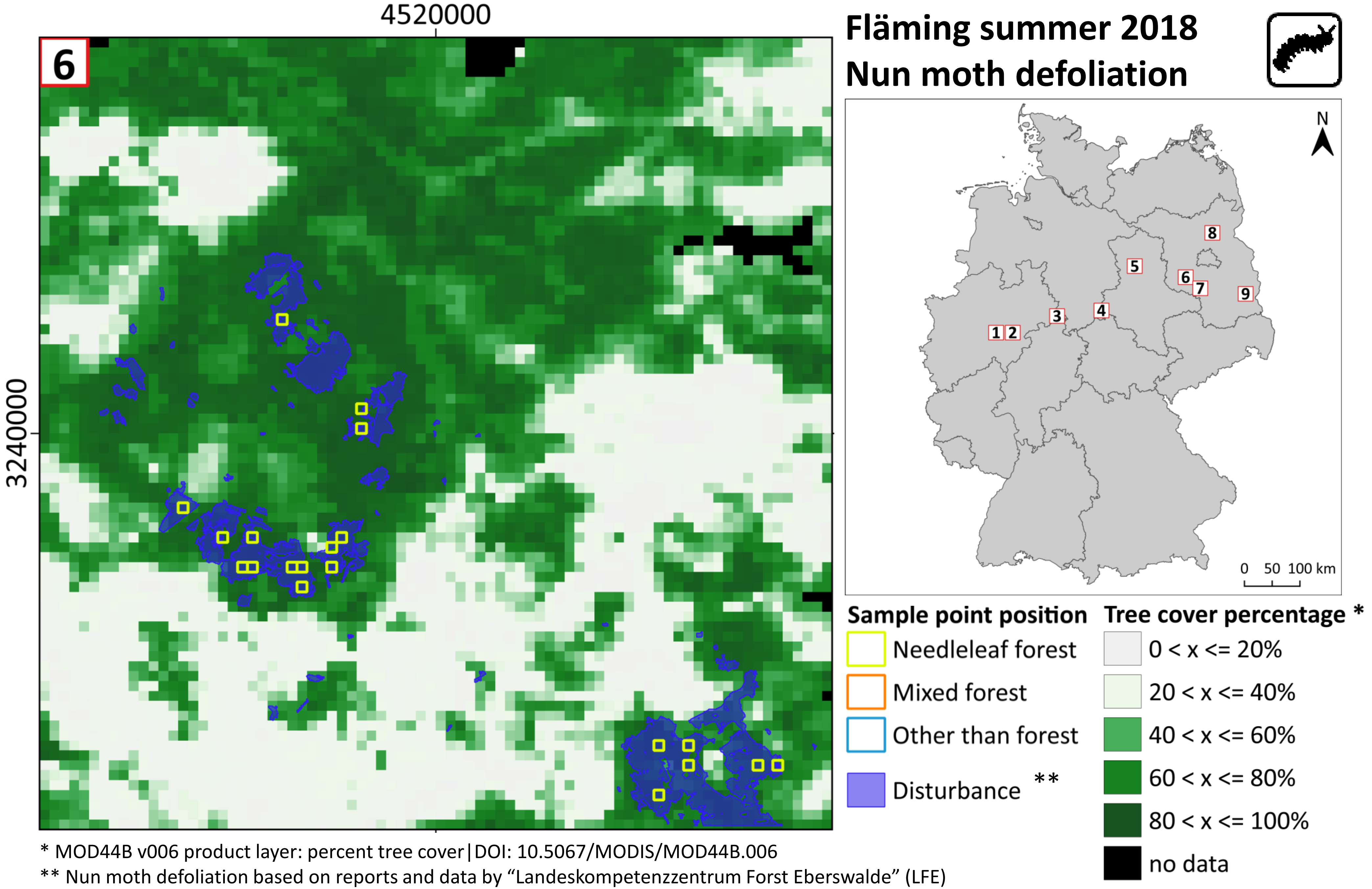

* MOD44B v006 product layer: percent tree cover|DOI: 10.5067/MODIS/MOD44B.006
** Nun moth defoliation based on reports and data by "Landeskompetenzzentrum Forst Eberswalde" (LFE)
https://mluk.brandenburg.de/cms/media.php/lbm1.a.3310.de/Waldzustandsbericht_BB_2018.pdf p.21

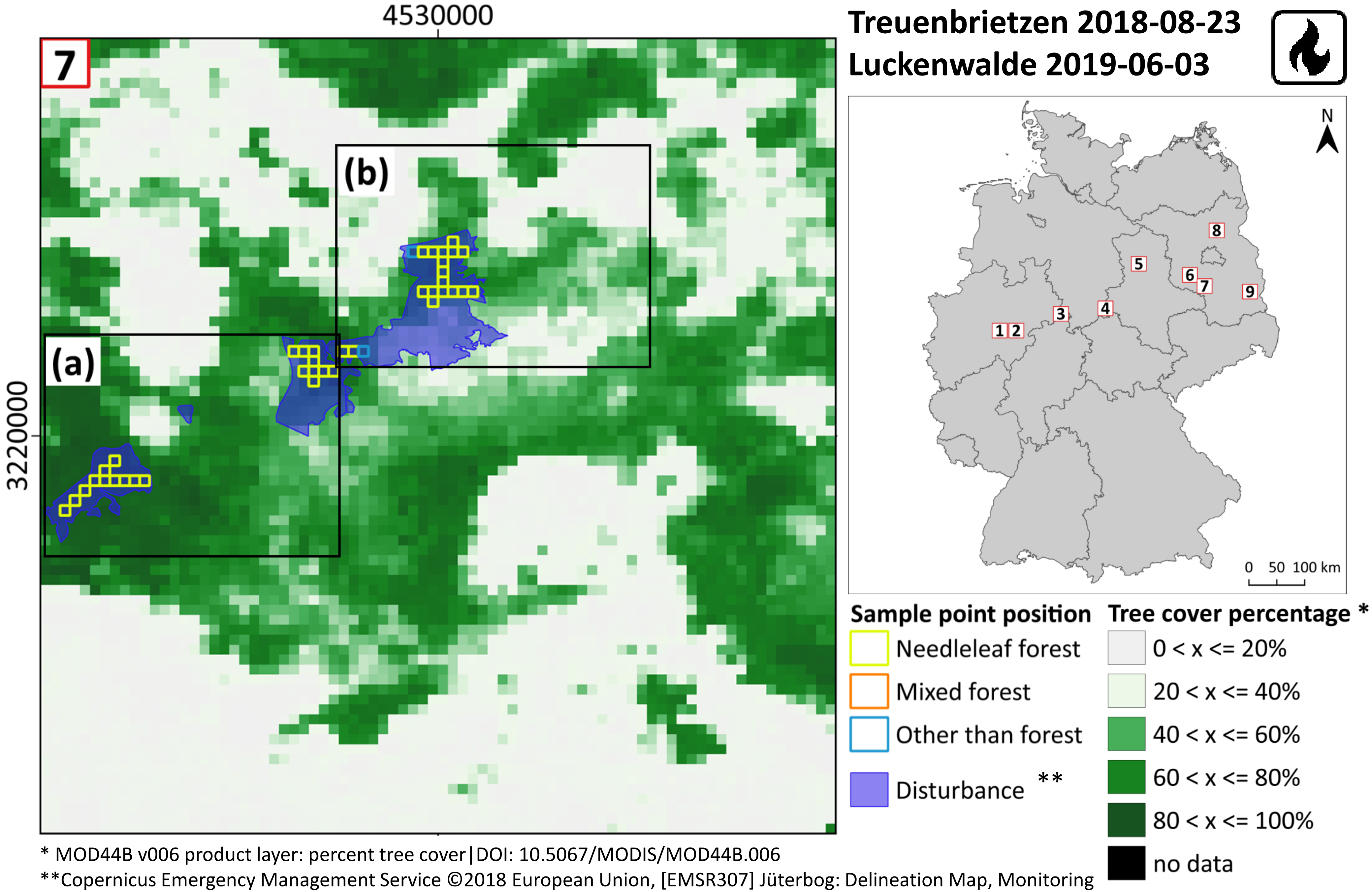

* MOD44B v006 product layer: percent tree cover|DOI: 10.5067/MODIS/MOD44B.006

**Copernicus Emergency Management Service ©2018 European Union, [EMSR307] Jüterbog: Delineation Map, Monitoring [illegible]

**Copernicus Emergency Management Service ©2019 European Union, [EMSR363] Luckenwalde: Delineation Map, Monitoring 3

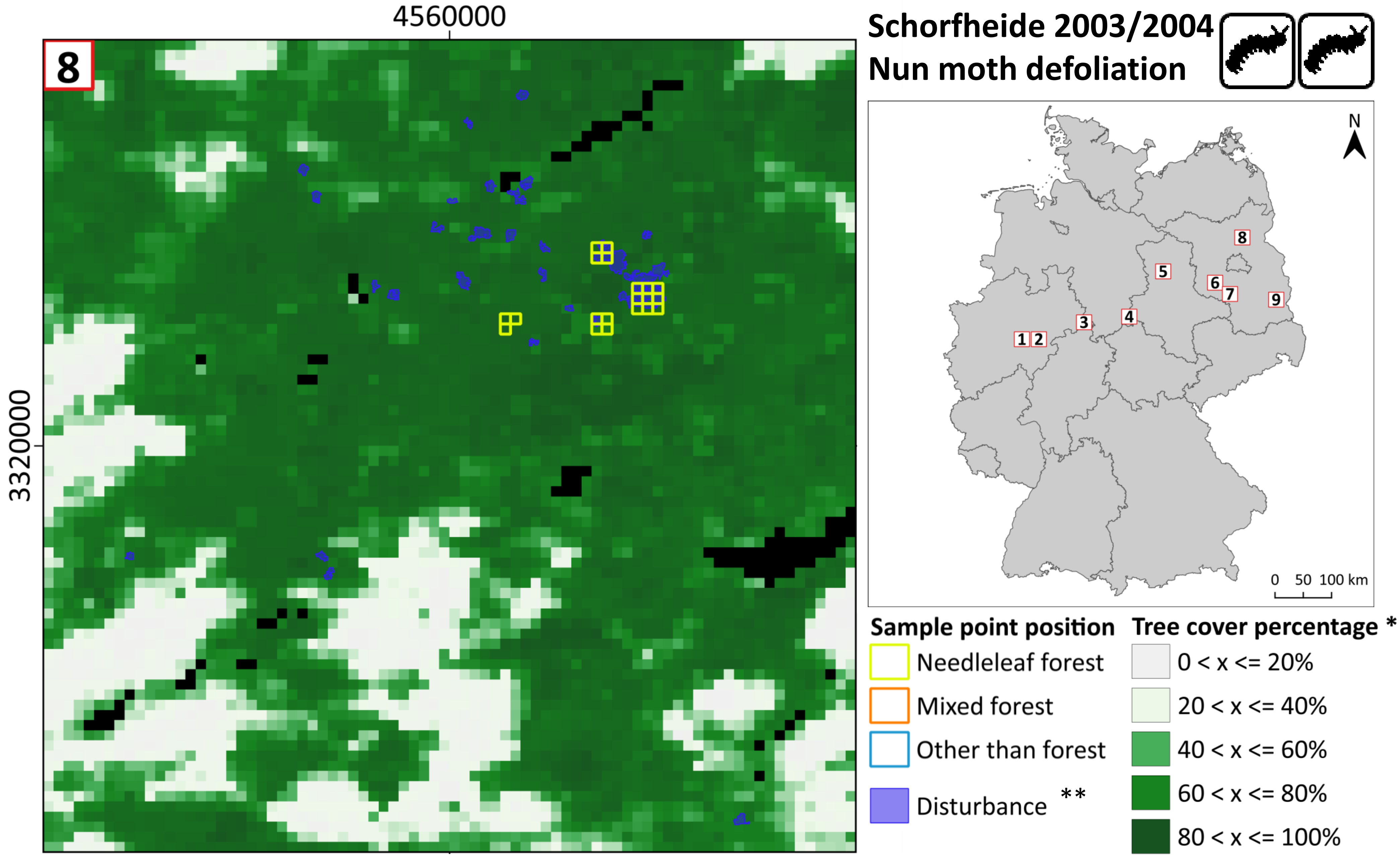

* MOD44B v006 product layer: percent tree cover|DOI: 10.5067/MODIS/MOD44B.006
https://forst.brandenburg.de/sixcms/media.php/9/bwin_verlust.pdf p. 41-42
**Silvicultural monitoring area for nun moth (*Lymantria monacha*) defoliation and recovery | WFS: http://www.Brandenburg-forst.e/geoserver/lfb1/wfs

Lieberose 2013/2014
Nun moth/Pine lappet

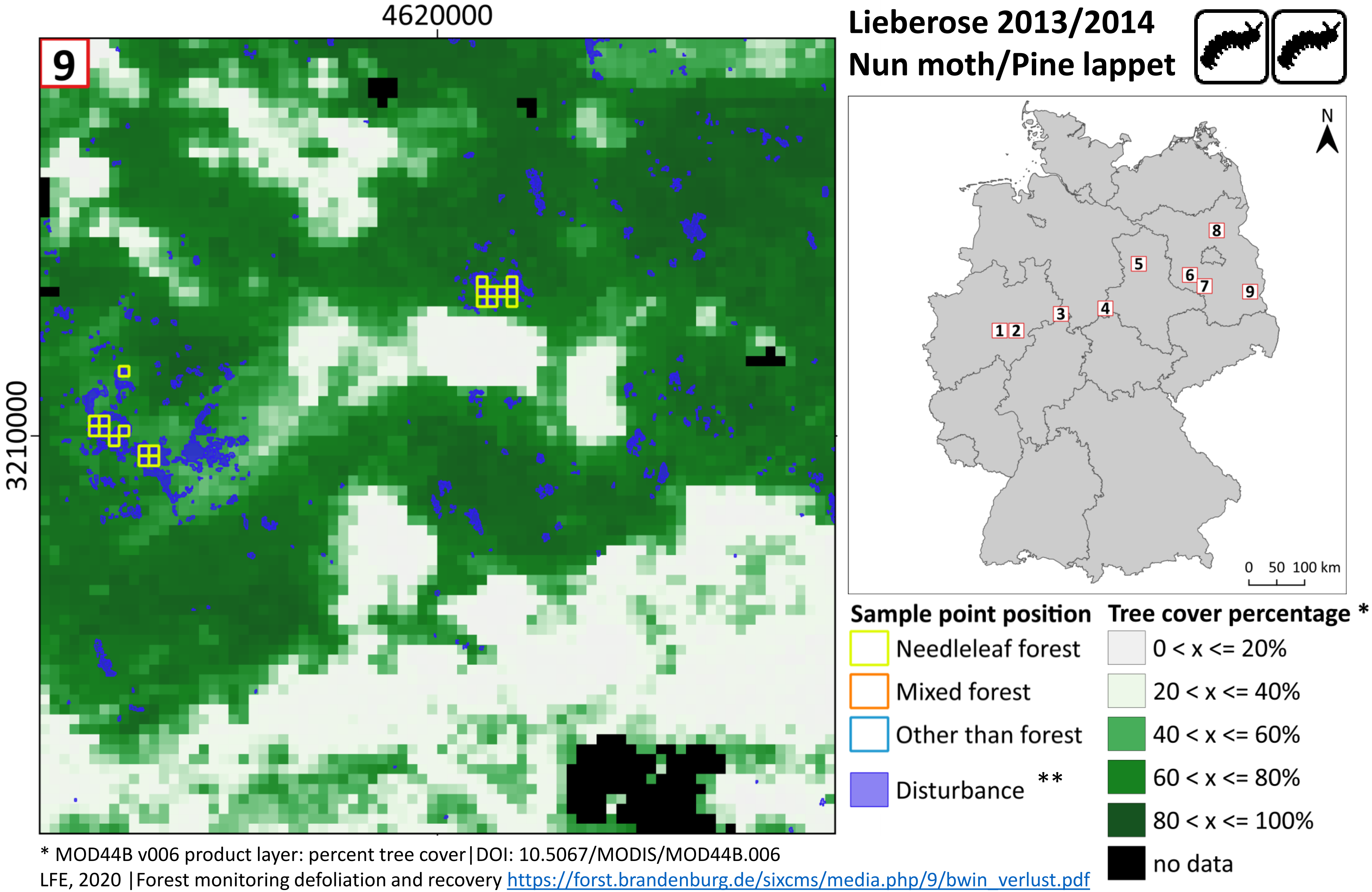

* MOD44B v006 product layer: percent tree cover|DOI: 10.5067/MODIS/MOD44B.006
LFE, 2020 |Forest monitoring defoliation and recovery https://forst.brandenburg.de/sixcms/media.php/9/bwin_verlust.pdf
**Hansen/UMD/Google/USGS/NASA | Global Forest Change 2000-2019 Forest Loss Year data available at: glad.earthengine.app/view/global-forest-change

Letzlingen 2009/2010
Pine sawfly/Diplodia

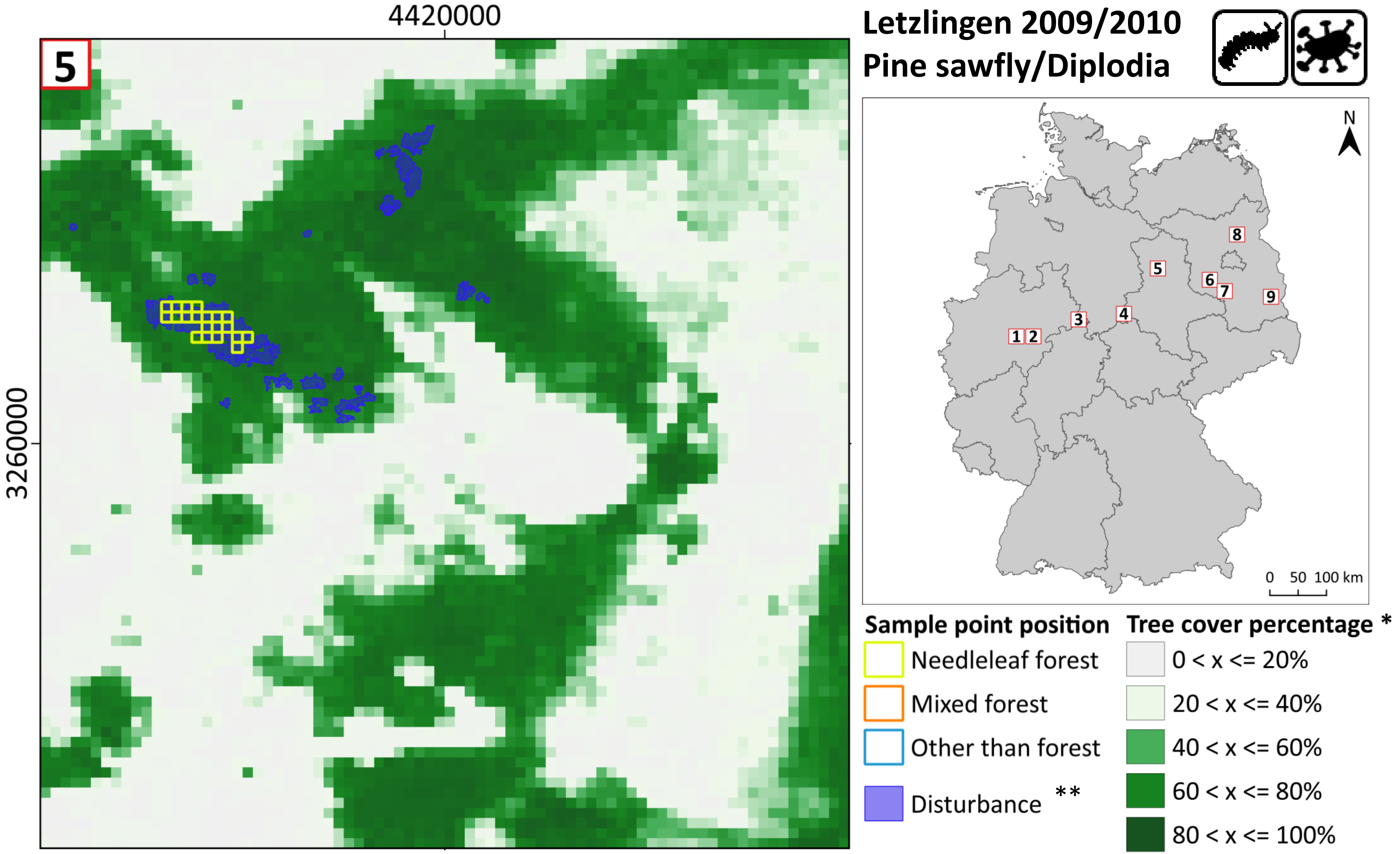

* MOD44B v006 product layer: percent tree cover|DOI: 10.5067/MODIS/MOD44B.006
https://www.nw-fva.de/fileadmin/user_upload/Verwaltung/Publikationen/2015/NWFVA_Bd13_2015_Klimawandel.pdf
**Hansen/UMD/Google/USGS/NASA | Global Forest Change 2000-2019 Forest Loss Year data available at: glad.earthengine.app/view/global-forest-change

Braunlage 2018-01-18
Wind storm “Friederike”

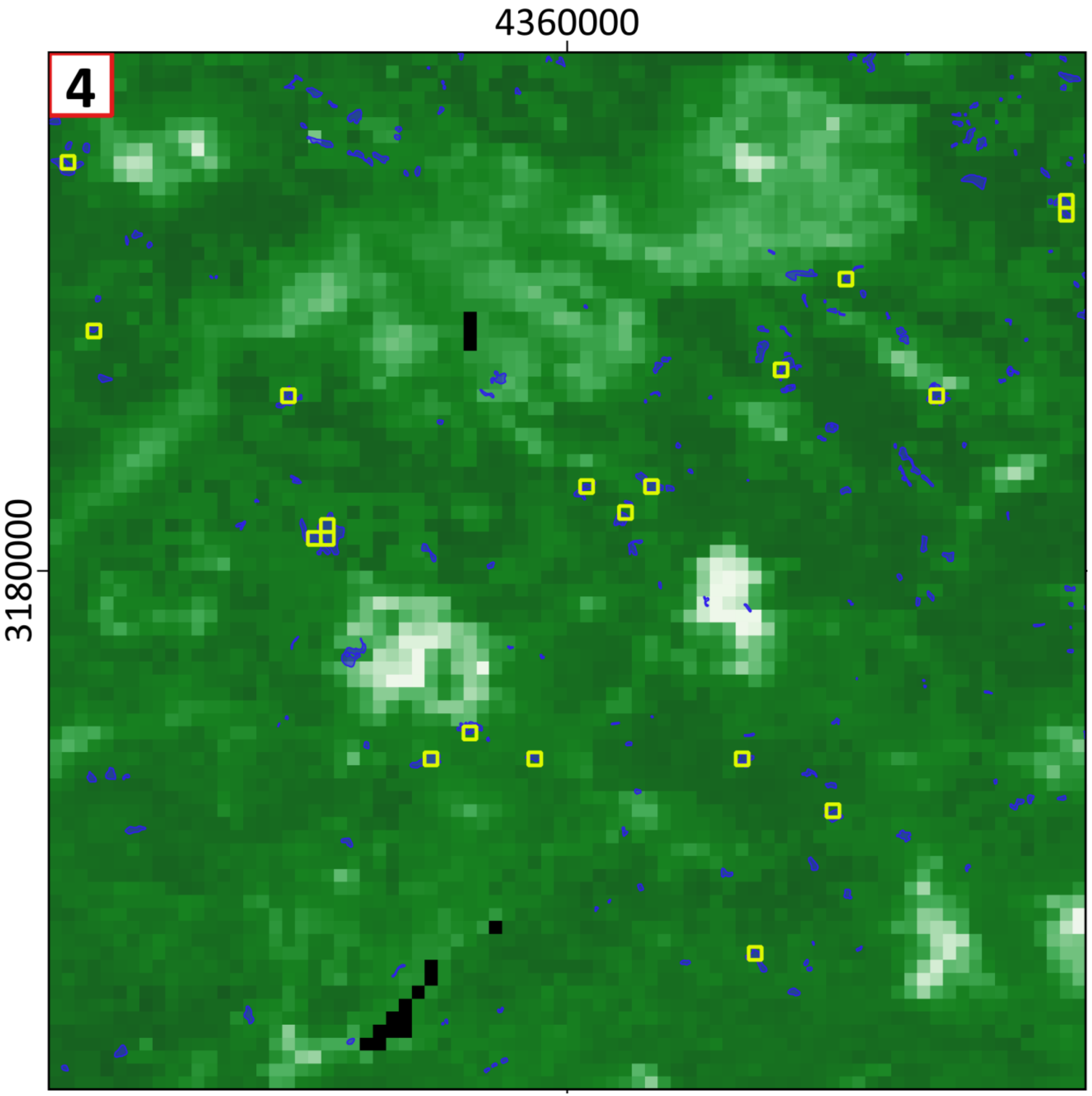

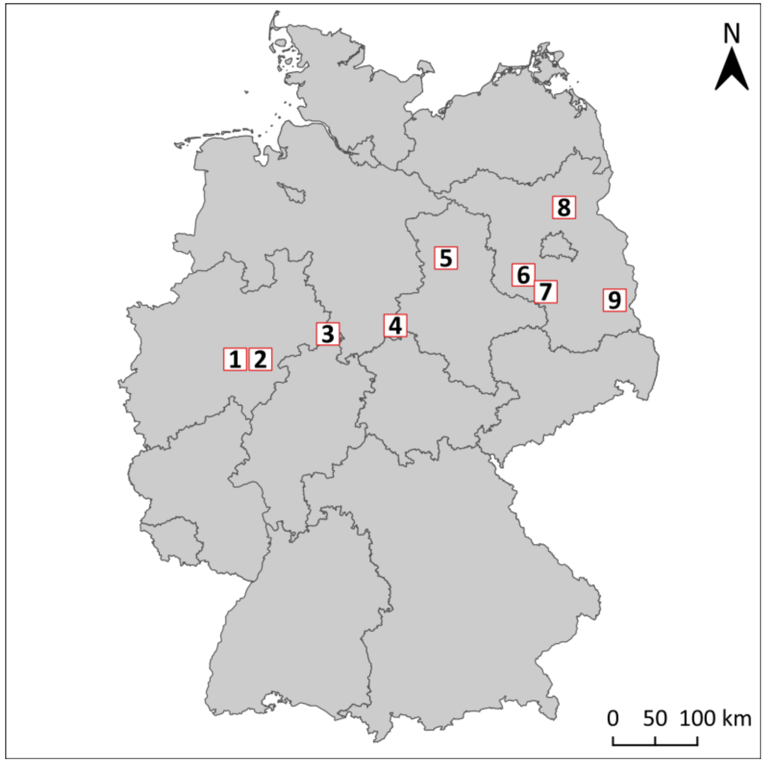

Sample point position

- Needleleaf forest
- Mixed forest
- Other than forest
- Disturbance **

Tree cover percentage *

- 0 < x <= 20%
- 20 < x <= 40%
- 40 < x <= 60%
- 60 < x <= 80%
- 80 < x <= 100%
- no data

* MOD44B v006 product layer: percent tree cover | DOI: 10.5067/MODIS/MOD44B.006
Copernicus Emergency Management Service © 2019 European Union, [EMSR266] Braunlage: Delineation Map, v1
**Land NRW, 2021 | https://www.opengeodata.nrw.de/produkte/umwelt_klima/wald_forst/wald/windwurfschadflaechen-friederike_EPSG25832_Shape.zip

Bad Karlshafen 2018-01-18
Wind storm “Friederike”

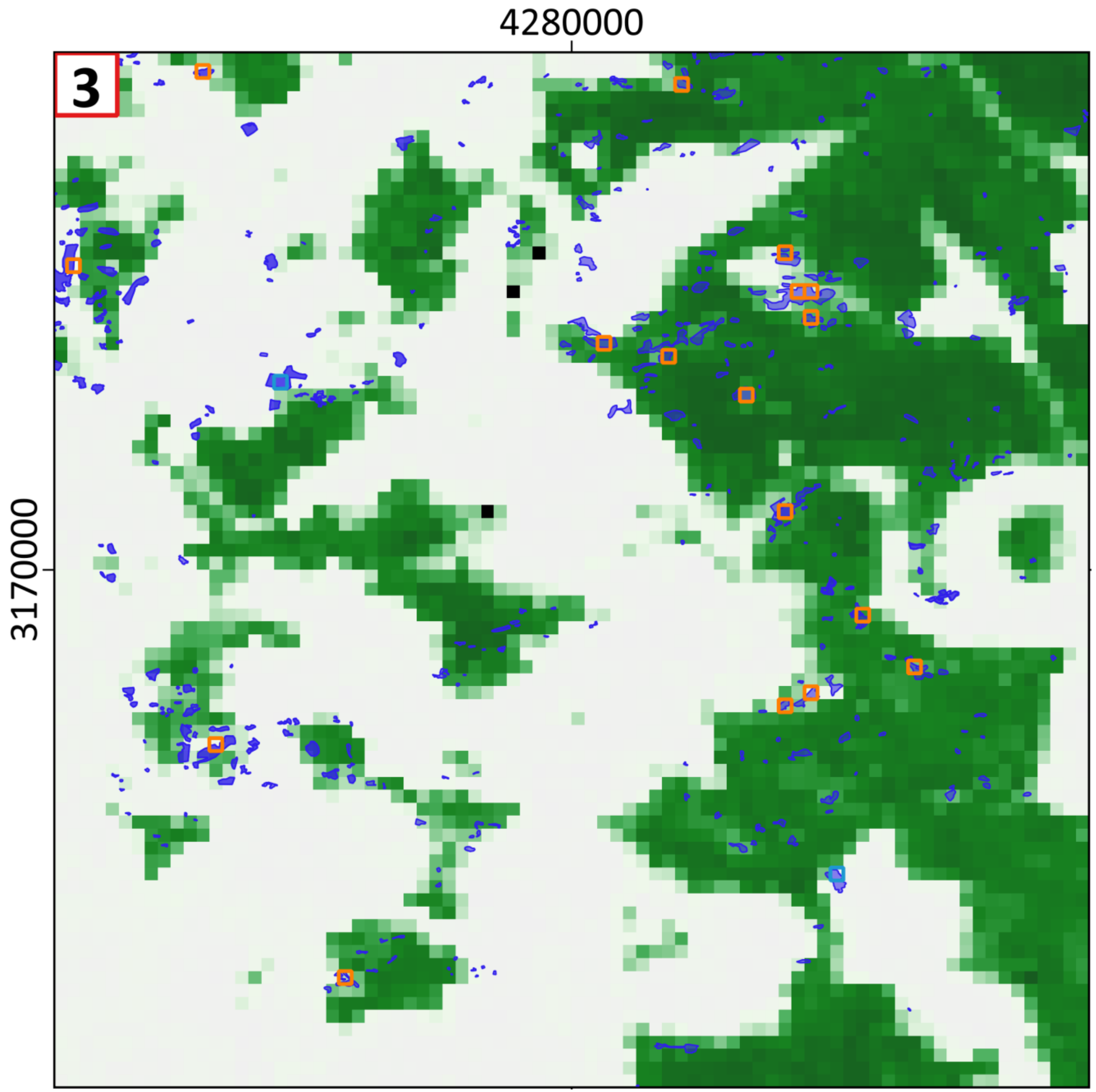

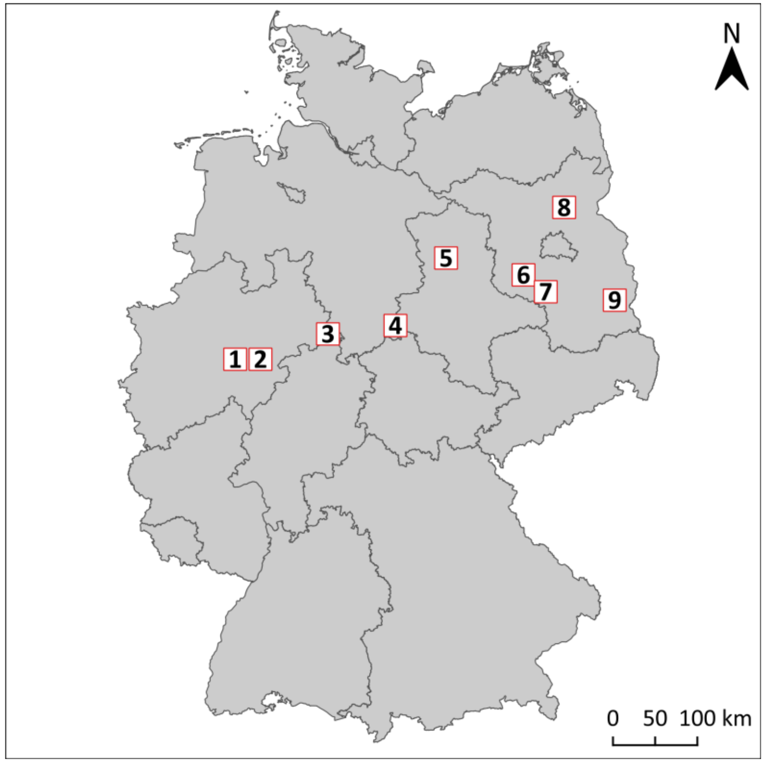

Sample point position

- Needleleaf forest
- Mixed forest
- Other than forest
- Disturbance **

Tree cover percentage *

- 0 < x <= 20%
- 20 < x <= 40%
- 40 < x <= 60%
- 60 < x <= 80%
- 80 < x <= 100%
- no data

* MOD44B v006 product layer: percent tree cover|DOI: 10.5067/MODIS/MOD44B.006

Copernicus Emergency Management Service © 2019 European Union, [EMSR266] Bad Karlshafen: Delineation Map, v1

**Land NRW, 2021 | https://www.opengeodata.nrw.de/produkte/umwelt_klima/wald_forst/wald/windwurfschadflaechen-friederike_EPSG25832_Shape.zip

Menschede 2007-01-18
Wind storm “Kyrill”

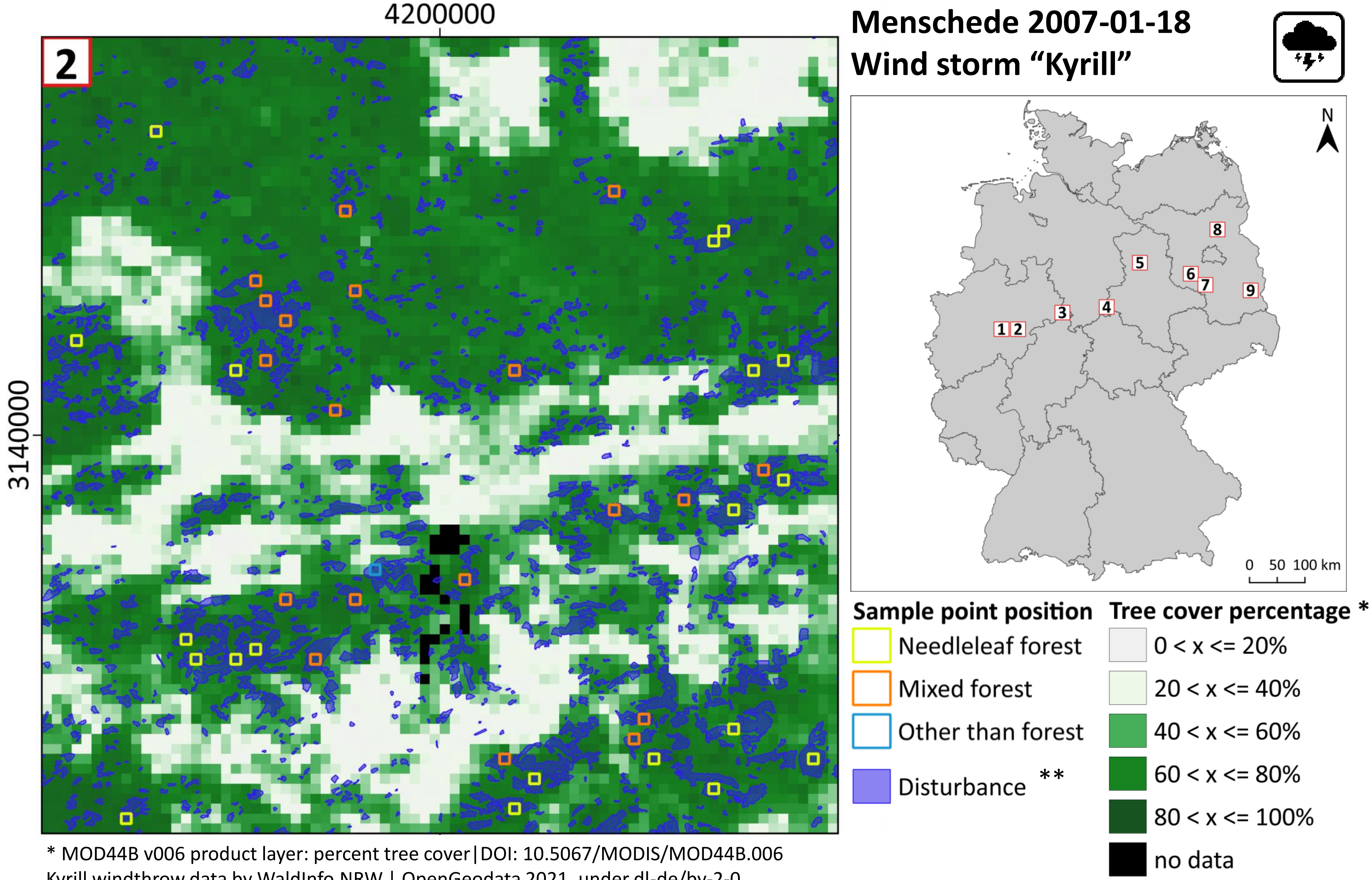

* MOD44B v006 product layer: percent tree cover|DOI: 10.5067/MODIS/MOD44B.006
Kyrill windthrow data by WaldInfo.NRW | OpenGeodata 2021, under dl-de/by-2-0
**Land NRW, 2021 | https://www.opengeodata.nrw.de/produkte/umwelt_klima/wald_forst/wald/windwurfschadflaechen-kyrill_EPSG25832_Shape.zip

NSG Hemer 2007-01-18
Wind storm “Kyrill”

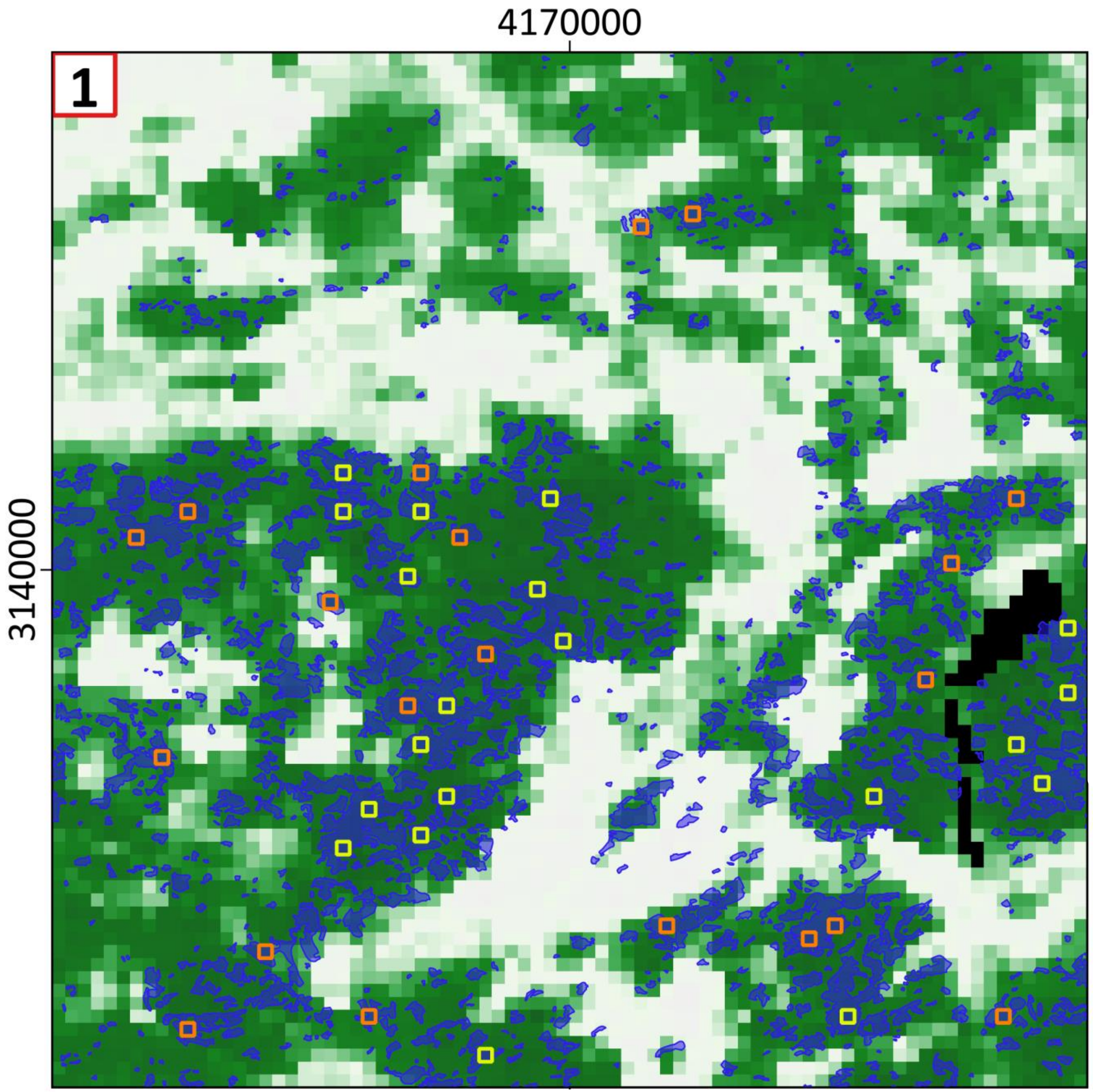

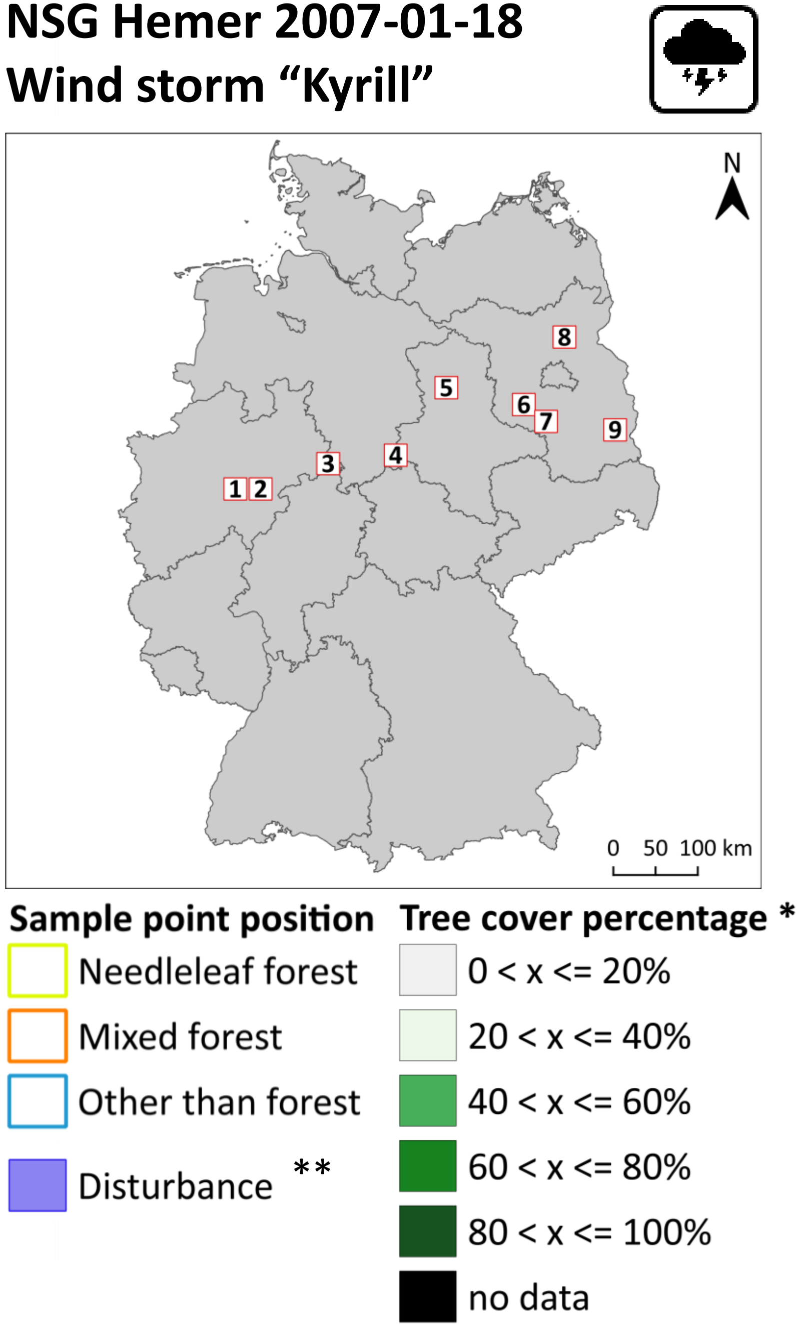

* MOD44B v006 product layer: percent tree cover|DOI: 10.5067/MODIS/MOD44B.006
Kyrill windthrow data by WaldInfo.NRW | OpenGeodata 2021, under dl-de/by-2-0
**Land NRW, 2021 | https://www.opengeodata.nrw.de/produkte/umwelt_klima/wald_forst/wald/windwurfschadflaechen-kyrill_EPSG25832_Shape.zip

decision tree

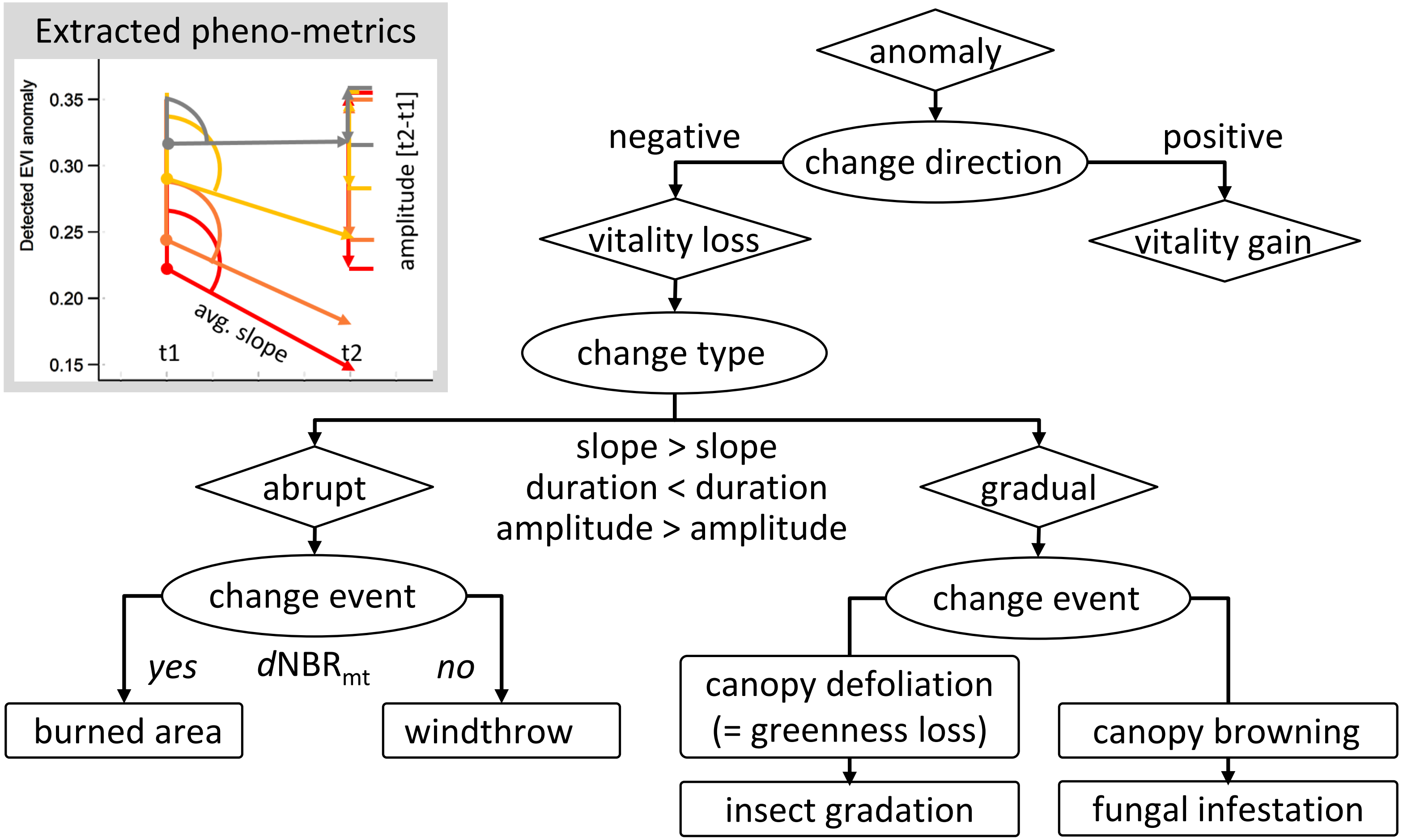

* dNBR$_{mt}$ = multi-temporal differenced Normalized Burn Ratio: Veraverbeke, S.; Lhermitte, S.; Verstraeten, W. W.; Goossens, R. (2011): A time-integrated MODIS burn severity assessment using the multi-temporal differenced normalized burn ratio (dNBR$_{mt}$). In *International Journal of Applied Earth Observation and Geoinformation* 13 (1), pp. 52–58. DOI: 10.1016/j.jag.2010.06.006.

“EXPERT KNOWLEDGE - INSECT DEFOLIATION”

Ergebnisse – Was beeinflusst die Massenvermehrung von Kiefernspinner, Nonne und Blattwespe?

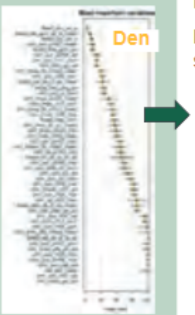

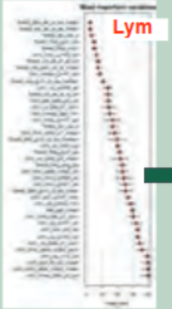

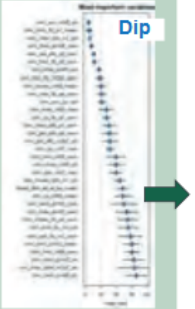

Kiefernspinner (Den):

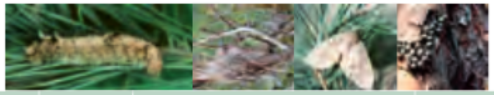

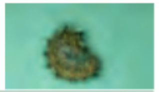

Die für die Klassifizierung von „Fraßabteilungen“ bedeutendsten Einflussfaktoren sind Bestandeskennwerte (*forest*), gefolgt von Klimakennwerten (*clim*).

Rang 1: ***forest_div_sw_e***
Rang 2: ***forest_sib_gym_al_ta_mean***
Rang 3: ***forest_div_sw_s***

→ Eine geringe Baumarten-Diversität in der Nachbarschaft (*sw* = Shannon-Index) leistet einen hohen Erklärungsanteil für Fraßereignisse durch den Kiefernspinner.

→ Die Gefährdung großflächiger Kiefernreinbestände (*gym*) durch Kieferngroßschädlinge ist bekannt und beruht auf einem üppigen Nahrungsangebot und einer geringen Habitateignung für natürliche Gegenspieler.

→ Die statistische Analyse deckt sich ebenfalls mit den Erfahrungen einer besonders hohen Gefährdung von Beständen geringerer Wuchsleistung (*sib* = rel. Bonität).

Januar	Februar-März	April - Mai	Juni - Juli - August			September - Oktober	November-Dezember
Überwinterung der Raupen im Boden	Aufbaumen der Raupen	Larven	Raupen/ Puppen	Puppen	Falter, Eier und L1	Raupen	Abbaumen in den Boden als L3/L4*

*Die seltene, 2 Jahre dauernde Entwicklung wurde nicht berücksichtigt

Rang 4: ***clim_sun_pm_05_mean*** → Wärme und Trockenheit im Mai fördern die Entwicklung der wechselwarmen Raupen. Sie können „ununterbrochen“ fressen. Wird das Puppenstadium schneller erreicht, sinkt u. a. die Angriffswahrscheinlichkeit von Larvenparasitoiden wie Raupenfliegen oder Schlupfwespen. Hohe Puppengewichte der Weibchen lassen hohe Eizahlen erwarten.

Rang 6: ***clim_sun_pm_09_sd*** →Warmes und trockenes Wetter im September begünstigt die Eilarvenentwicklung, senkt die Mortalität dieses in der Regel empfindlichsten Larvenstadiums.

Rang 9: ***clim_tmax_fly_m2p2_mean*** → Es ist bekannt, das Wärme und Trockenheit während des Falterfluges Kopulation und Eiablage begünstigen, damit letztlich auch die Eizahl.

Rang 10: ***clim_sun_m02_mean*** → Hohe Sonneneinstrahlung im Februar erwärmt den Boden und beschleunigt das Aufbaumen der Raupen, damit sind die Raupen eher sicher vor natürlichen Gegenspielern wie Mäusen, Schwarzwild, Pilzen oder Bakterien.

Rang 15: ***clim_tmax_m10_sum*** → Ein warmer Oktober begünstigt die Fraßaktivität der Raupen und damit das Anlegen von Energiereserven für die Überwinterungsphase im Boden (in Fettkörper und larvalen Speicherproteinen).

Nonne (Lym):

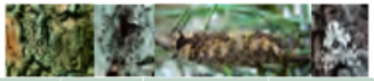

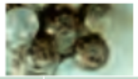

Die Datenanalyse belegt für diese Art den entscheidenden Einfluss der Waldeigenschaften.

Rang 1-5, 7, 8, 10: ***forest...***

→ Großflächige, mittelalte Kiefernreinbestände in „eintöniger“ Nachbarschaft fördern noch stärker als beim Kiefernspinner Massenvermehrungen.

→ Es wird deutlich, dass Waldumbaumaßnahmen das Schadgeschehen der Nonne nachhaltig beeinflussen. Die Erhöhung der Diversität bei den Baumarten scheint hier maßgeblich.

Januar – Februar - März	April	Mai - Juni	Juli	August - September	Oktober- November - Dezember
Ei	Ei, L1	Raupen	Puppe, Falter	Falter, Eiräupchenentwicklung	Ei

Rang 6: ***clim_tmax_pm09_sd*** → Schwenke (1978) beschreibt drei Phasen der Eilarvenentwicklung. Der Embryonalentwicklung folgt im September eine Phase der Konstituierung, die mit entscheidend für das Überleben der Larven im Winter ist.

Rang 20: ***clim_vp_pm04_sd*** → Das Vertrocknen der Eilarven im Ei kann häufige Mortalitätsursache sein. Trockenheit im April (*vp* – Dampfdruck), kurz vor Schlupf der Räupchen, erhöht die Wahrscheinlichkeit.

Gemeine Kiefernbuschhornblattwespe (Dip):

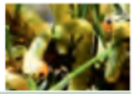

Hier ist der Einfluss von Klimafaktoren (Rang 1-21: *clim*...) sehr markant. Nur bei geeigneter Witterung entwickelt sich im Sommer eine 2. Generation. Deren Herbstfraß ist oft bestandesbedrohend.

November - März	April-Mai	Juni	Juli	August	September- Oktober	November - Dezember
Überwinterung im Kokon im Boden	Wespe, Ei	Larve	Kokon, wenn 2 Generationen	Eiablage und L1 (2. Generation)	Larven (2. Generation)	Kokon

Rang 1: ***clim_sun_m08_sd*** → Die Junglarven der 2. Generation profitieren von einem sonnigen August.
Rang 2: ***clim_tmin_fly_p1_mean*** → Eine geringe Temperatur nach der Flugperiode der Wespen könnte das Eintrocknen der Eigelege reduzieren.

Weiterführende Literatur:
Möller, K., Hentschel, R., Wenning, A.; Schröder, J. (2017): Improved Outbreak Prediction for Common Pine Sawfly (*Diprion pini* L.) by Analyzing Floating ‘Climatic Windows’ as Keys for Changes in Voltinism. *Forests* 2017, *8*(9), 319; doi:10.3390/f8090319
Hentschel, R.; Möller, K.; Wenning, A.; Degenhardt, A.; Schröder, J. (2018): Importance of Ecological Variables in Explaining Population Dynamics of Three Important Pine Pest Insects. Front. Plant Sci. 9: 1667; https://doi.org/10.3389/fpls.2018.01667

Ausblick

- Da die Hauptschadgebiete von Nonne und Kiefernspinner bekannt sind, lässt sich ableiten, wo Waldumbaumaßnahmen besonders große Effekte auf die Häufigkeit und Intensität von Massenvermehrungen haben können. Für den Landeswald sind solche Karten in Vorbereitung (Hentschel 2019).
- Rechenroutinen, um mit Hilfe der aktuellen Witterung Schadprognosen zu unterstützen, sind Ziel neuer Projektanträge.
- Eine komplexe Waldschutzdatenbank ist komfortabler und erhoffter „Nebeneffekt“.

Dr. Katrin Möller, Dr. Rainer Hentschel , Dr. Jens Schröder , Aline Wenning, Dr. Annett Degenhardt (Kontakt: Katrin.Moeller@LFB.Brandenburg.de)
Landeskompetenzzentrum Forst Eberswalde, Alfred-Möller-Str. 1, 16225 Eberswalde

Source: https://forst.brandenburg.de/sixcms/media.php/9/efs67.pdf, *S.94*
Möller, Katrin; Hentschel, Rainer; Wenning, Aline; Schröder, Jens (2017): Improved Outbreak Prediction for Common Pine Sawfly (Diprion pini L.) by Analyzing Floating ‘Climatic Windows’ as Keys for Changes in Voltinism. In Forests 8 (9), p. 319. DOI: 10.3390/f8090319. https://doi.org/10.3390/f8090319
Hentschel, Rainer; Möller, Katrin; Wenning, Aline; Degenhardt, Annett; Schröder, Jens (2018): Importance of Ecological Variables in Explaining Population Dynamics of Three Important Pine Pest Insects. In *Front. Plant Sci.* 9, p. 1667. DOI: 10.3389/fpls.2018.01667. https://doi.org/10.3389/fpls.2018.01667
